# A Reduced F_420_-Dependent Nitrite Reductase in an Anaerobic Methanotrophic Archaeon

**DOI:** 10.1128/jb.00078-22

**Published:** 2022-06-13

**Authors:** Christian Heryakusuma, Dwi Susanti, Hang Yu, Zhou Li, Endang Purwantini, Robert L. Hettich, Victoria J. Orphan, Biswarup Mukhopadhyay

**Affiliations:** a Genetics, Bioinformatics, and Computational Biology Ph.D. Program, Virginia Tech, Blacksburg, Virginia, USA; b Department of Biochemistry, Virginia Tech, Blacksburg, Virginia, USA; c Division of Geological and Planetary Sciences, California Institute of Technology, Pasadena, California, USA; d Biosciences Division, Oak Ridge National Laboratory, Oak Ridge, Tennessee, USA; e Virginia Tech Carilion School of Medicine, Virginia Tech, Blacksburg, Virginia, USA; University of Florida

**Keywords:** anaerobic methane oxidation, anaerobic methanotrophic archaea, methanogen, F_420_-dependent nitrite reductase, F_420_-dependent sulfite reductase, FsrI, FsrII, coenzyme F_420_, F_420_H_2_, deazaflavin, electron transfer, iron-sulfur cluster, methane

## Abstract

Anaerobic methanotrophic archaea (ANME), which oxidize methane in marine sediments through syntrophic associations with sulfate-reducing bacteria, carry homologs of coenzyme F_420_-dependent sulfite reductase (Fsr) of Methanocaldococcus jannaschii, a hyperthermophilic methanogen from deep-sea hydrothermal vents. *M. jannaschii* Fsr (*Mj*Fsr) and ANME-Fsr belong to two phylogenetically distinct groups, FsrI and FsrII, respectively. *Mj*FsrI reduces sulfite to sulfide with reduced F_420_ (F_420_H_2_), protecting methyl coenzyme M reductase (Mcr), an essential enzyme for methanogens, from sulfite inhibition. However, the function of FsrIIs in ANME, which also rely on Mcr and live in sulfidic environments, is unknown. We have determined the catalytic properties of FsrII from a member of ANME-2c. Since ANME remain to be isolated, we expressed ANME2c-FsrII in a closely related methanogen, Methanosarcina acetivorans. Purified recombinant FsrII contained siroheme, indicating that the methanogen, which lacks a native sulfite reductase, produced this coenzyme. Unexpectedly, FsrII could not reduce sulfite or thiosulfate with F_420_H_2_. Instead, it acted as an F_420_H_2_-dependent nitrite reductase (FNiR) with physiologically relevant *K_m_* values (nitrite, 5 μM; F_420_H_2,_ 14 μM). From kinetic, thermodynamic, and structural analyses, we hypothesize that in FNiR, F_420_H_2_-derived electrons are delivered at the oxyanion reduction site at a redox potential that is suitable for reducing nitrite (E^0^′ [standard potential], +440 mV) but not sulfite (E^0^′, −116 mV). These findings and the known nitrite sensitivity of Mcr suggest that FNiR may protect nondenitrifying ANME from nitrite toxicity. Remarkably, by reorganizing the reductant processing system, Fsr transforms two analogous oxyanions in two distinct archaeal lineages with different physiologies and ecologies.

**IMPORTANCE** Coenzyme F_420_-dependent sulfite reductase (Fsr) protects methanogenic archaea inhabiting deep-sea hydrothermal vents from the inactivation of methyl coenzyme M reductase (Mcr), one of their essential energy production enzymes. Anaerobic methanotrophic archaea (ANME) that oxidize methane and rely on Mcr, carry Fsr homologs that form a distinct clade. We show that a member of this clade from ANME-2c functions as F_420_-dependent nitrite reductase (FNiR) and lacks Fsr activity. This specialization arose from a distinct feature of the reductant processing system and not the substrate recognition element. We hypothesize FNiR may protect ANME Mcr from inactivation by nitrite. This is an example of functional specialization within a protein family that is induced by changes in electron transfer modules to fit an ecological need.

## INTRODUCTION

Microbial degradation of complex biopolymers in the marine sediments annually generates 85 to 300 Tg of methane, a potent greenhouse gas (1). However, oceans contribute a minor fraction to atmospheric emissions, and this is largely due to a microbial process known as the anaerobic oxidation of methane (AOM) (1, 2). In early 2000s, this conversion was shown to be a syntrophic process, where methane oxidation performed by environmental anaerobic methanotrophic archaea (ANME) is coupled to sulfate reduction by sulfate-reducing bacteria (2–4). ANME are polyphyletic, with several clades closely related to methanogenic archaea in the phylum *Halobacteriota* (5). Collectively, these anaerobes employ a reversed methanogenesis pathway for methane oxidation (2, 6). Over the last 2 decades, several mechanisms for the syntrophy in AOM have been put forward, and it remains an active research topic (2–4, 7–13). In one case, it was hypothesized that ANME-2 archaea are capable of directly reducing sulfate to oxidize methane (3), bringing new attention to the genomic data linked to sulfate metabolism in ANME, including the homologs of F_420_-dependent sulfite reductase (Fsr) (8, 14–18). Fsr was first observed in Methanocaldococcus jannaschii, a representative of an ancient lineage of methanogens from deep-sea hydrothermal vents (19). Genetic and biochemical analyses support a role in sulfite detoxification by the *M. jannaschii* Fsr (*Mj*Fsr), converting this potent inhibitor of methyl coenzyme M reductase, an essential enzyme for the methanogens, to sulfide, an essential sulfur source for anabolism in this hyperthermophilic methanogen (19–21). All described hyperthermophilic methanogenic archaea from deep-sea hydrothermal vents carry the *fsr* gene, suggesting that the ability to transform sulfite into bioavailable sulfur is advantageous in this environment (19, 22). It is also present in several hyperthermophilic, thermophilic, and mesophilic methanogens from hot springs, sewage digesters, peatland, and hypersaline and marine sediments (14, 19, 22). However, the specific function of Fsr in these diverse archaea has not been confirmed.

While early studies on Fsr identified homologs of the protein in ANME (22, 23), observations made in the field studies and laboratory experiments and further bioinformatic analyses brought about an ANME-specific focus on this enzyme (14–16, 24). An environmental metaproteomic analysis of marine methane seep sediment showed that a homolog of Fsr in ANME-2 lineages is overexpressed in the environment (14–16, 24). On the other hand, in sediment microcosm experiments, the members of ANME-2c are seen as sensitive to sulfite (14), and SO_4_^2−^ utilization in ANME-2 is likely assimilatory in nature and not coupled to methane oxidation (15). These findings raised new possibilities for the function of Fsr in ANME, and a comparative primary structure analysis has brought further attention to this topic (14).

The enzyme Fsr is a two-domain protein, where the N-terminal F_420_H_2_ dehydrogenase unit (Fsr-N) retrieves reducing equivalents from F_420_H_2_ and the C-terminal dissimilatory sulfite reductase (Dsr) unit (Fsr-C) utilizes them to reduce sulfite to sulfide (19, 22). A phylogenetic analysis showed that *Mj*Fsr-C and ANME-Fsr-C are distinct in their primary structures, and accordingly, the respective homologs have been named FsrI and FsrII (14). With one exception, all FsrI homologs occur in certain methanogens that belong to phylum *Methanobacteriota*, and the organisms carrying FsrII homologs belong to the phylum *Halobacteriota* (6, 14); Methanohalobium evestigatum, a moderate thermophile from *Halobacteriota* that was isolated from a salt lagoon, carries both FsrI and FsrII (14). Taken together, these studies suggest that an FsrII likely contributes to the ecophysiology of ANME in a manner that is different from the role of FsrI in *M. jannaschii*, and hence, FsrI and FsrII could have distinct catalytic properties. Accordingly, we have characterized the structural and catalytic properties of a homogenous preparation of an FsrII of the ANME-2c lineage, ANME2c-FsrII-6D, and found that it acts as an F_420_-dependent nitrite reductase (FNiR) and is incapable of reducing sulfite with F_420_H_2_. We describe the thermodynamic and structural basis for this distinction and discuss the possible ecophysiological relevance of the enzyme.

## RESULTS

### Heterologous expression and purification of recombinant ANME2c-FsrII-6D.

A recombinant Methanosarcina acetivorans strain carrying pDS701, a replicable expression vector for ANME2c-FsrII, was constructed for the study. An extract of cells of this strain that were induced with tetracycline was able to catalyze sulfite-dependent oxidation of reduced methyl viologen. This activity was absent in native *M. acetivorans* extracts, which lack Fsr (14, 19, 22). Thus, it was concluded that ANME2c-FsrII-6D was expressed with activity in *M. acetivorans*(pDS701) under tetracycline induction. Similar to *Mj*FsrI (19), the ANME2c-FsrII-6D activity in the cell extracts was highly oxygen sensitive. Based on this initial finding, all enzyme purification steps were performed under strictly anaerobic conditions.

The enzyme was purified to apparent homogeneity via ammonium sulfate precipitation followed by sequential phenyl-Sepharose, QAE-Sephadex, and F_420_-Sepharose chromatography steps (19). It did not bind to the QAE-Sephadex at the operating pH of 7, and the same result was obtained with DEAE-Sephadex. However, the anion exchanger bound and removed other negatively charged non-FsrII proteins and molecules that could have affected the performance of the F_420_ affinity-based purification step that followed. This negative purification step was useful for our purpose. The chromatography results also suggested that at pH 7, ANME2c-FsrII-6D either carried a net positive charge or was neutral. An opposite scenario exists for *Mj*FsrI, which binds to QAE-Sephadex at pH 7 (19) and therefore carries a negative charge under the operating conditions. A typical purification experiment yielded 0.72 ± 0.30 mg ANME2c-FsrII-6D protein per gram (wet weight) of cell pellet of the recombinant *M. acetivorans* strain.

**FIG 1:**
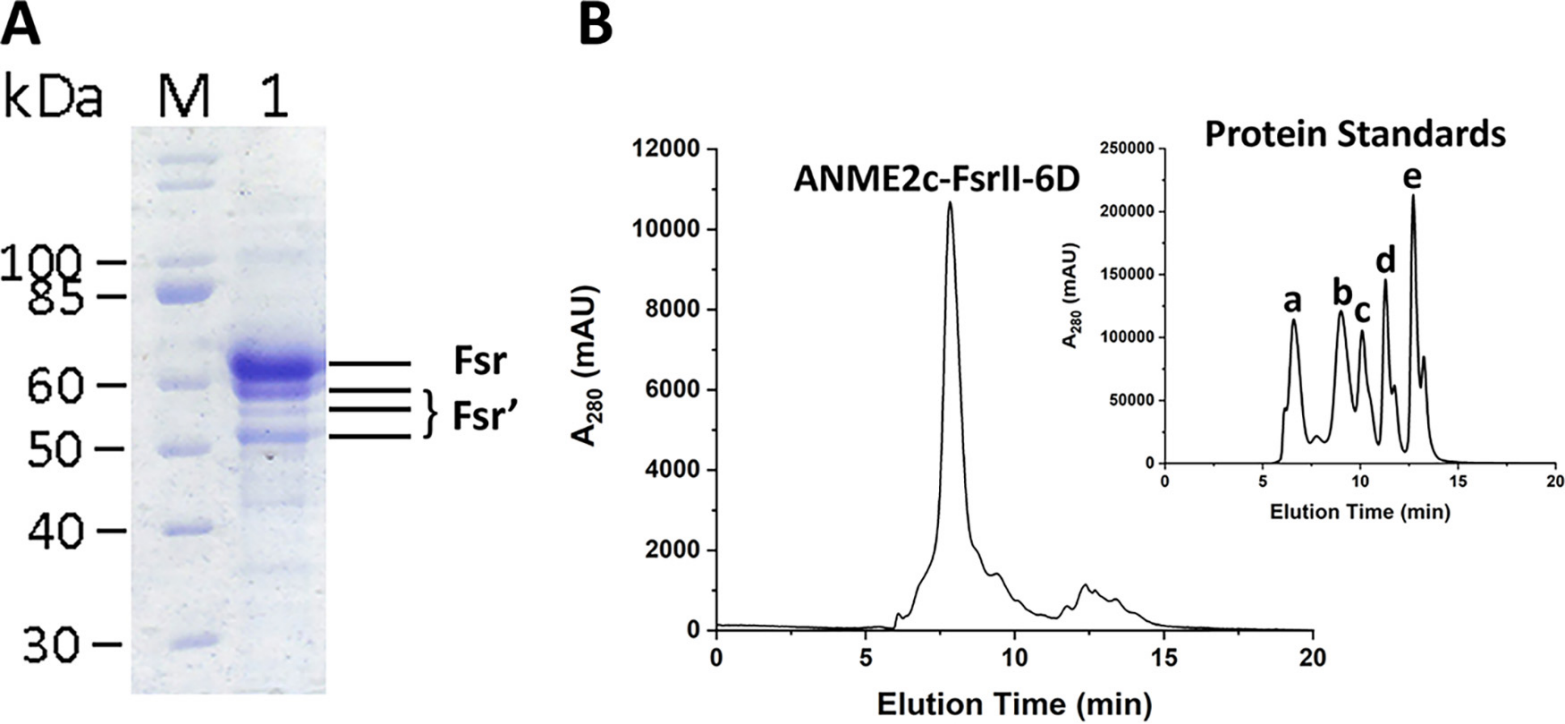
Analysis for subunit size and quaternary structure of ANME2c-FsrII-6D. (A) SDS-PAGE gel with F_420_ affinity-purified recombinant ANME2c-FsrII-6D. Lanes: M, protein ladder; 1, ANME2c-FsrII-6D preparation. Fsr, ~70 kDa, intact FsrII; Fsr′, ~52, ~55, and ~58 kDa degradation products of FsrII. (B) Size exclusion-chromatographic analysis of ANME2c-FsrII-6D. The largest peak corresponded to Fsr (20 μg protein). (Inset) Elution of the following calibration standards (catalog number 151-1901; Bio-Rad, Hercules, CA): a, thyroglobulin, 670,000 Da; b, gamma globulin, 158,000 Da; c, ovalbumin, 44,000 Da; d, myoglobin, 17,000 Da; e, vitamin B_12_, 1,350 Da. Figure S1 shows the calibration plot.

In SDS-PAGE, the preparation obtained from the F_420_-Sepharose chromatography step exhibited four bands (Fig. 1A) at ~52, ~55, ~58, and ~70 kDa, of which the last one matched the theoretical subunit size of ANME2c-FsrII-6D (69.23 kDa). Mass-spectrometric analyses confirmed that each of the bands corresponded to FsrII, indicating that the ~70 kDa band represented the intact subunit, with the smaller Fsr fragments representing degradation products likely produced during the sample preparation. A similar observation has been reported for *Mj*FsrI (19). Confirming that the preparation of ANME2c-FsrII-6D obtained from the F_420_ affinity chromatography step was homogeneous, we continued with structural and spectroscopic characterization of the recombinant Fsr protein.

### Structural and spectroscopic characteristics of ANME2c-FsrII-6D.

**FIG 2:**
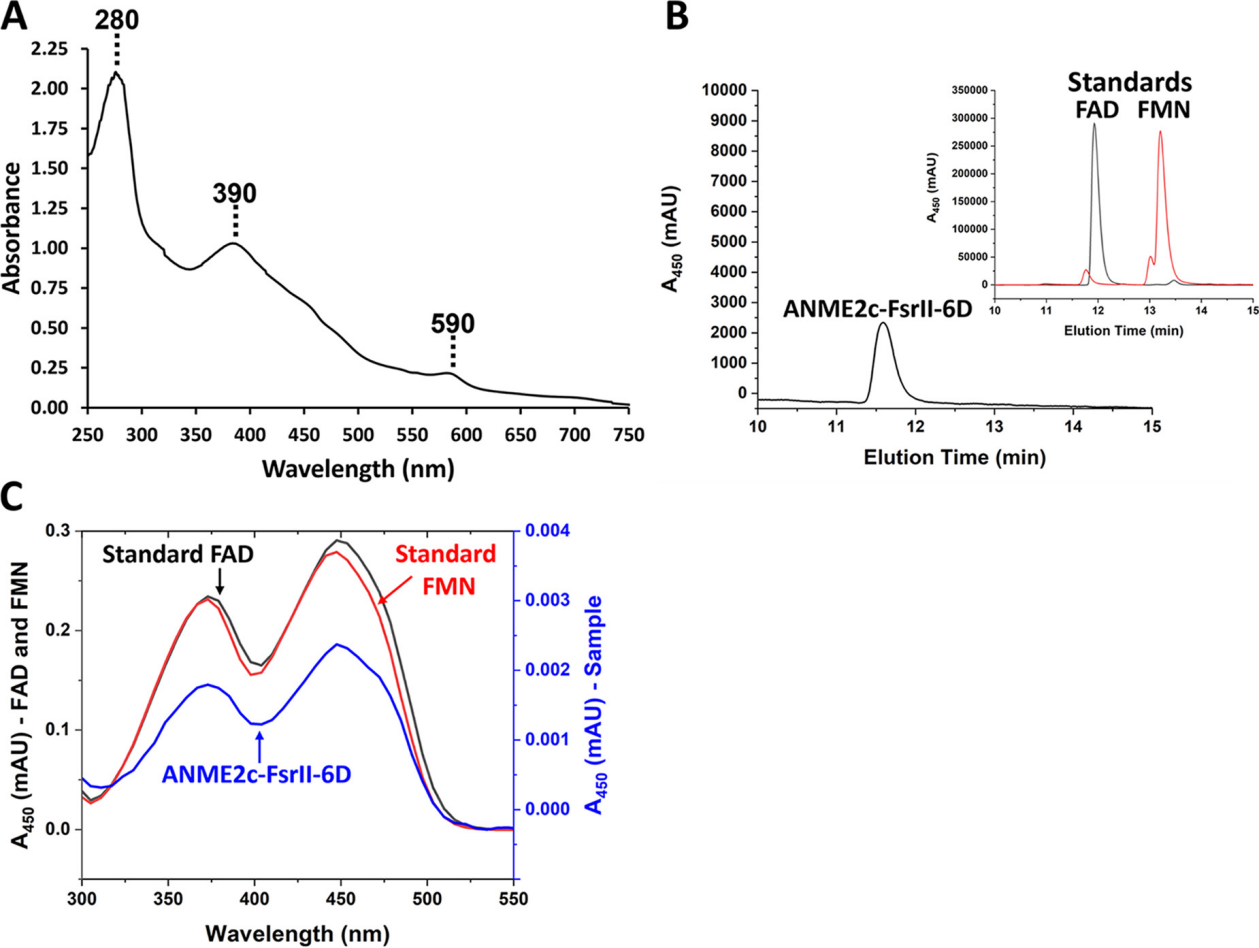
Analysis for prosthetic groups of ANME2c-FsrII-6D. (A) UV-visible spectrum of FsrII. A 300-μL anaerobic solution of 84 μg of homogeneous ANME2c-FsrII-6D in 100 mM potassium phosphate buffer (pH 7) containing 250 mM NaCl was analyzed in a quartz cuvette with a 1-cm light path sealed with a no. 00000 rubber stopper (EPDM rubber stopper, WidgetCo, Houston, TX). (B) Reverse-phase HPLC analysis of a methanol-methylene chloride extract of ANME2c-FsrII-6D. (Main plot) Analysis of a 100-μL methanol-methylene chloride extract of 63.2 μg homogeneous protein. The peak corresponds to FAD. (Inset) Analysis of a 10-μL solution of 1 mM FAD and FMN in distilled water. (C) UV-visible spectrum of flavin cofactor extracted from ANME2c-FsrII-6D and resolved on a reverse-phase column as described for panel B.

From the size exclusion chromatography data, the apparent native molecular mass of ANME2c-FsrII-6D was determined to be 289.44 kDa (Fig. 1B; also, see Fig. S1 in the supplemental material). This value and the subunit size as determined via SDS-PAGE (Fig. 1A) indicated that ANME2c-FsrII-6D was a tetramer of ~70 kDa subunits. The UV-visible spectrum of ANME2c-FsrII-6D exhibited three peaks at 280, 390, and 590 nm (Fig. 2A), which are typical of siroheme in the low-spin ferric state (19, 25). A reversed phase high-performance liquid chromatography (HPLC) analysis of a methanol-methylene chloride extract of the protein showed that purified recombinant ANME2c-FsrII-6D contained FAD (Fig. 2B and C); the elution time and the UV-visible spectrum of the eluting cofactor were identical to those of FAD. An estimation based on HPLC analysis showed that 63.2 μg or 0.91 nmol of ANME2c-FsrII-6D contained 1.08 nmol FAD, suggesting that a subunit of ANME2c-FsrII-6D carried one bound molecule of this flavin.

Bathophenanthroline and methylene blue assays revealed that ANME2c-FsrII-6D contained 15.36 ± 1.97 mol of iron and 15.02 ± 2.07 mol of acid-labile sulfur per subunit. These results suggested that each subunit of ANME2c-FsrII-6D assembled four [Fe_4_-S_4_] clusters.

### Catalytic properties of ANME2c-FsrII-6D. (i) Nitrite and hydroxylamine reduction.

**FIG 3:**
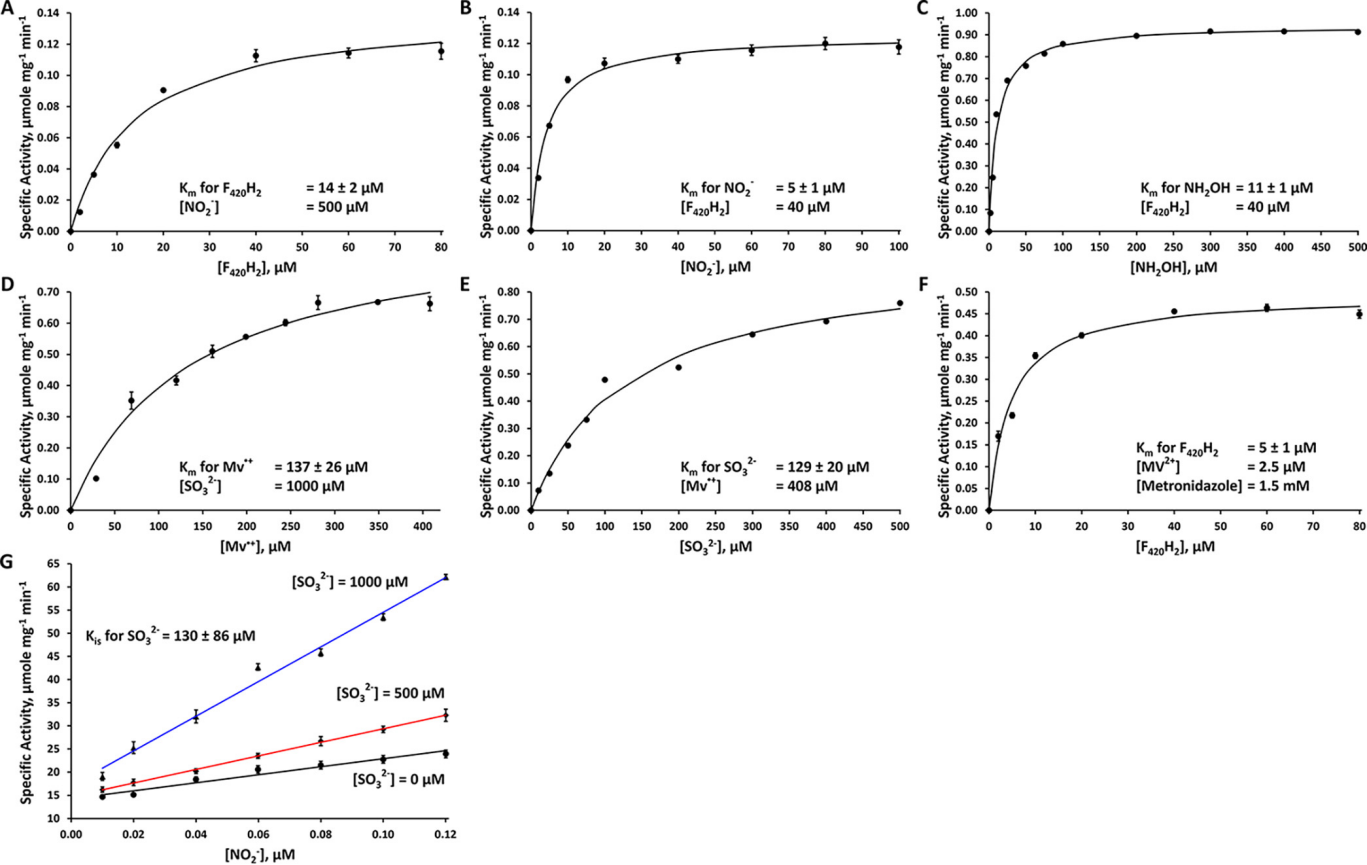
Kinetic analysis of ANME2c-FsrII-6D reactions. (A to F) Specific activities of the enzyme at various concentrations of F_420_H_2_ (A), nitrite (B), hydroxylamine (C), reduced methyl viologen (D), sulfite (E), and F_420_H_2_-oxidized methyl viologen-metronidazole (F). (G) Double reciprocal plots for the data on the inhibition of nitrite reductase activity by sulfite. Each data point is an average of values from three assays. In panels A to F, each solid curve represents the best fit of the data to the Henry-Michaelis-Menten hyperbola function, *v* = *V*_max_ [S]/(*K_m_* + [S]). Specific activity is defined as micromoles of F_420_H_2_ or reduced methyl viologen oxidized per minute per milligram of enzyme. A value for the amounts of sulfite or nitrite consumed was taken as one-third or one-sixth of that for F_420_H_2_ or reduced methyl viologen, respectively, assuming that sulfide or ammonia was the sole product. For panel G, the data were analyzed by fitting to the model *v* = *V*_max_ [S]/{*K_m_* (1+[I]/*K_i_*) + [S]}.

The lack of sulfite reduction activity with F_420_H_2_, the presumed native electron donor for Fsr, was unexpected, and further experiments were conducted to determine whether ANME2c-FsrII-6D was capable of reducing other substrates with F_420_H_2_. Notably, in assays with both nitrite and hydroxylamine, a substantial F_420_H_2_ oxidation activity was observed; hydroxylamine is a common intermediate of enzymatic reduction of nitrite (26–31). Furthermore, the addition of 0.5 mM dithiothreitol (DTT) stimulated the nitrite and hydroxylamine reduction activity by 1.5- and 5-fold, respectively. Thus, further assays of these activities occurred in the presence of DTT. At a fixed concentration of 500 μM for nitrite and a concentration range of 1 to 80 μM for F_420_H_2_, the apparent *K_m_* for F_420_H_2_ was determined to be 14 ± 2 μM and the maximum velocity (*V*_max_) value was 0.2 ± 0.01 μmol of F_420_H_2_ oxidized or 0.3 ± 0.02 μmol electrons transferred per min per mg enzyme (Fig. 3A). Similarly, with 40 μM F_420_H_2_ and 2 to 100 μM nitrite, the apparent *K_m_* for nitrite was found to be 5 ± 1 μM and the *V*_max_ was 0.2 ± 0.01 μmol of F_420_H_2_ oxidized or 0.3 ± 0.01 μmol electrons transferred per min per mg enzyme (Fig. 3B). The apparent *K_m_* for hydroxylamine was also determined. Assays at 40 μM for F_420_H_2_ and 2 to 500 μM hydroxylamine, the apparent *K_m_* for hydroxylamine was found to be 11 ± 1 μM and the *V*_max_ was 1 ± 0.02 μmol of F_420_H_2_ oxidized or 2 ± 0.04 μmol electrons transferred per min per mg enzyme (Fig. 3C). These kinetic parameter values are also shown in Table 1. Considering that the reduction of nitrite to ammonium (NO_2_^−^ to NH_4_^+^) is a 6-electron process and that for hydroxylamine to ammonium (NH_2_OH to NH_4_^+^) is a 2-electron process, ANME2c-FsrII-6D reduced hydroxylamine 21 times faster than nitrite. In a reaction mixture with 0.08 μmol of F_420_H_2_ and 0.40 μmol of nitrite, ANME2c-FsrII-6D produced 0.02 ± 0.001 μmol of F_420_ and 0.006 ± 0.005 μmol of ammonia after 30 min of reaction; these values represent averages from three independent assays. Considering that 3 mol of F_420_H_2_ (6 electrons) would be needed to reduce 1 mol of nitrite to ammonia, the above values represent 90% recovery of reducing equivalents from F_420_H_2_ into ammonia, indicating that it was the sole product. ANME2c-FsrII-6D was not able to utilize NADH or NADPH as electron donors with nitrite or hydroxylamine as an oxidant. The second partial reaction of the nitrite reductase activity could not be assayed because nitrite chemically oxidized the electron source, reduced methyl viologen (MV^·+^); the first partial reaction did not involve nitrite, and the respective activity was assayed as described in the following section. The *k*_cat_ values for the nitrite reductase activity with respect to F_420_H_2_ and nitrite were 19 and 18 min^−1^, respectively, and for the sulfite reductase activity it was 64 min^−1^ with respect to both HSO_3_- and MV^·+^ (Table 1). A turnover value of 130 min^−1^ was recorded for the hydroxylamine reductase activity with respect to NH_2_OH (Table 1).

**TABLE 1:** Kinetic parameters of ANME2c-FsrII-6D^*a*^

Substrate concentrations	*K_m_* (μM) for the varied substrate	*K*_cat_ (min^−1^)	*k*_cat_/*K_m_* (μM^−1^ min^−1^) (with respect to the varied substrate)	Reaction (electron donor)
NO_2_^−^, 500 μM; F_420_H_2_, varied (1–80 μM)	14 ± 2 (F_420_H_2_)	19 ± 1	2 ± 0.3 (F_420_H_2_)	NO_2_^−^ reduction (F_420_H_2_)
F_420_H_2_, 40 μM; NO_2_^−^, varied (2–100 μM)	5 ± 1 (NO_2_^−^)	18 ± 1	4 ± 1 (NO_2_^−^)	NO_2_^−^ reduction (F_420_H_2_)
F_420_H_2_, 40 μM; NH_2_OH, varied (2–500 μM)	11 ± 1 (NH_2_OH)	130 ± 3	12 ± 2 (NH_2_OH)	NH_2_OH reduction (F_420_H_2_)
HSO_3_^−^, 1,000 μM; MV^·+^, varied (29–408 μM)	137 ± 26 (MV^·+^)	64 ± 5	0.5 ± 0.1 (MV^·+^)	HSO_3_^−^ reduction (MV^·+^)^*b*^
MV^·+^, 408 μM; HSO_3_^−^, varied (10–500 μM)	129 ± 20 (HSO_3_^−^)	64 ± 4	0.5 ± 0.1 (HSO_3_^−^)	HSO_3_^−^ reduction (MV^·+^)^*b*^

a *K_m_*, Michaelis-Menten constant; *k*_cat_, turnover number expressed in terms of number of electrons transferred per enzyme subunit per min; *k*_cat_/*K_m_*, catalytic efficiency with respect to the varied substrate.

b Nitrite reduction activity could not be assayed with MV^·+^ as the electron donor because NO_2_^−^ oxidizes MV^·+^ chemically.

### (ii) Sulfite reduction.

We tested the ability of ANME2c-FsrII-6D to reduce relevant sulfur oxyanions with the reductants: F_420_H_2_ (the predicted native electron donor), NADH, and NADPH. ANME2c-FsrII-6D did not oxidize F_420_H_2_ with sulfite or thiosulfate as electron acceptors and similar results were obtained in assays with NADH and NADPH as reductants. These conclusions were based on activity assays both with and without 0.5 mM DTT and more than 10 independently generated purified preparations.

Upon finding a lack of sulfite reduction with F_420_H_2_ by the enzyme, we examined if ANME2c-FsrII-6D could catalyze the following two partial reactions that have been observed with *Mj*FsrI (19): F_420_H_2_ oxidation, transfer of electrons from F_420_H_2_ to the protein bound flavin coenzyme as catalyzed by Fsr-N; sulfite reduction, utilization of electrons transferred from reduced flavin via iron-sulfur clusters and siroheme prosthetic group of Fsr-C (19, 22). While the first is assayed with methyl viologen (MV^2+^) as the direct electron acceptor, the second partial reaction is observed with reduced methyl viologen (MV^·+^) as the reductant (19). The ANME2c enzyme catalyzed both partial reactions. The second reaction did not occur with thiosulfate as the electron acceptor.

We calculated the *K_m_* value for the substrates and maximum velocity (*V*_max_) for sulfite reduction from the second partial reaction. In assays with a fixed concentration of sulfite (1 mM) and a concentration range of 29 to 408 μM for MV^·+^, the apparent *K_m_* for MV^·+^ was determined to be 137 ± 26 μM and the value of maximum velocity (*V*_max_) was 1 ± 0.1 μmol of MV^·+^ oxidized or 1 ± 0.1 μmol electrons transferred per min per mg enzyme (Fig. 3D). Similarly, at a fixed concentration of 408 μM for MV^·+^ and a sulfite concentration range of 10 to 500 μM, the apparent *K_m_* for sulfite was found to be 129 ± 20 μM and the *V*_max_ value was 1 ± 0.1 μmol of MV^·+^ oxidized or the same amounts of electrons transferred per min per mg enzyme (Fig. 3E). If DTT was omitted from the assay, the apparent *K_m_* for sulfite dropped to 28 ± 5 μM and the *V*_max_ value dropped to 0.3 ± 0.01 μmol of MV^·+^ oxidized, or 0.3 ± 0.01 μmol electrons transferred per min per mg enzyme. In a reaction mixture with 0.8 μmol of MV^·+^ and 0.8 μmol of sulfite, ANME2c-FsrII-6D produced 0.02 ± 0.01 μmol of sulfide after 30 min of reaction; these values represented averages from three independent assays. This result represents a utilization of 13% of the reductant (MV^·+^) supplied in the assay toward sulfide production from sulfite, which is a 6-electron reduction process. The enzyme was able to oxidize F_420_H_2_ with a mixture of methyl viologen (MV^2+^) and metronidazole, where the former served as the direct artificial electron acceptor for the enzyme and was reduced to MV^·+^, and metronidazole regenerated MV^2+^ from MV^·+^. From assays with 2.5 μM MV^2+^, 1.5 mM metronidazole, and a concentration range of 2 to 80 μM for F_420_H_2_, the apparent *K_m_* for F_420_H_2_ was determined to be 5 ± 1 μM and *V*_max_ was 0.5 ± 0.02 μmol of F_420_H_2_ oxidized or 1 ± 0.04 μmol electrons transferred per min per mg enzyme (Fig. 3F). A summary of the enzyme’s kinetic parameters is shown in Table 1.

### (iii) Inhibition of nitrite reductase activity by sulfite.

The pattern seen in the double-reciprocal or Lineweaver-Burk plots of the data collected at a fixed concentration of 40 μM for F_420_H_2_ and with nitrite at 8.3 to 100 μM and sulfite at three different concentrations, 0, 500, and 1000 μM, suggested that sulfite was a competitive inhibitor of ANME2c-FsrII-6D for the enzyme’s nitrite reduction activity (Fig. 3G). In this assay, sulfite was not reduced, as ANME2c-FsrII-6D cannot reduce this oxyanion with F_420_H_2_, and truly acted as an inhibitor. A fitting of the data to the competitive inhibition model yielded a value of 130 ± 86 μM for the apparent inhibition constant (*K_i_*) for sulfite; the standard error for the fitting suggested that the inhibition did not follow a standard model, and the mechanism underlying this departure is currently unknown.

### Structural features of ANME2c-FsrII-6D and other FsrIIs and FsrIs—analysis of primary structure and computational models for three-dimensional structures.

Since the N- and C-terminal halves of an Fsr (Fsr-N and Fsr-C) represent two distinct domains performing two parts of the overall reactions, F_420_H_2_ oxidation and sulfite or nitrite reduction, respectively, we analyzed the features for ANME2c-FsrII-6D-N and ANME2c-FsrII-6D-C separately. From a comparison with *Mj*FsrI, where the residues 1 to 311 and 325 to 620 represent the N- and C-terminal domains (19, 22), the ANME2c-FsrII-6D-N and ANME2c-FsrII-6D-C units were assigned to the 1 to 341 and 356 to 642 segments of ANME2c-FsrII.

### N-terminal domain—iron-sulfur clusters.

**FIG 4:**
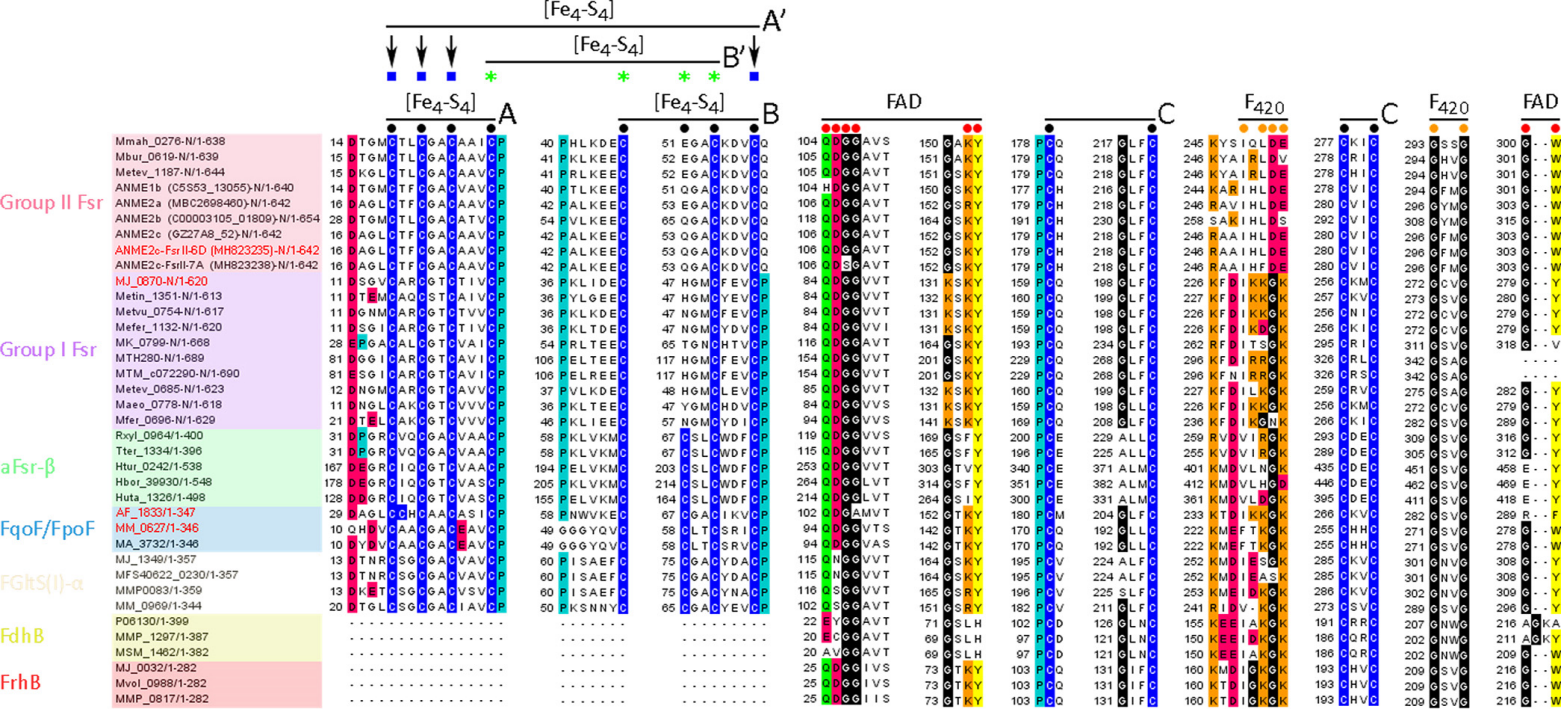
Multiple primary sequence alignment for ANME2c-FsrII-6D-N homologs. Conserved amino acid residues are represented as follows: blue, cysteine; cyan, proline; black, glycine; red, negatively charged (D or E); green, polar uncharged (S, T, N, or Q); orange, positively charged (H, R, or K); yellow, aromatic (F, Y, or W). Black bullets with overlines and A and B notations show [Fe_4_-S_4_] cluster assembly motifs; black circles with overlines and C notation represent conserved cysteine residues, red circles with overlines show the flavin binding motif (89, 90), and orange circles with overlines show the F_420_ binding motif (89); blue squares and green stars with overlines and A′ and B′ notations, respectively, show [Fe_4_-S_4_] cluster assembly motifs identified in the AlphaFold2 models. Fsr, F_420_-dependent sulfite reductases (14, 19, 22); aFsr-β, F_420_H_2_ dehydrogenase subunit of a putative F_420_H_2_-dependent assimilatory type siroheme sulfite reductase (22); FqoF/FpoF, F_420_H_2_ dehydrogenase subunit of a membrane-bound proton pumping F_420_H_2_ dehydrogenase complex (32, 33); FGltS(I)-α, F_420_H_2_ dehydrogenase subunit of a putative F_420_H_2_-dependent glutamate synthase (22, 91); FdhB, formate dehydrogenase subunit B (92); FrhB: F_420_-reducing [NiFe]-dehydrogenase subunit B (77). Open reading frame (ORF) numbers of the proteins, except those from ANME, are presented in the NCBI format (abbreviated organism name_respective ORF number). For an ANME protein, the NCBI ORF number appears within parentheses following the abbreviated organism name. “-N” indicates that only the N-terminal part of the polypeptide is shown. Ranges following slashes are amino acid residues for the complete protein. The names of proteins that are of particular interest in the study are shown in red. ANME2c-FsrII-6D-N, MH823235; *Mj*FsrI-N, MJ_0870; FpoF of Archaeoglobus fulgidus, AF_1833; FqoF of Methanosarcina mazei, MM_0627. Abbreviations of organism names: Mmah, Methanohalophilus mahii DSM 5219; Mbur, Methanococcoides burtonii DSM 6242; Metev, Methanohalobium evestigatum Z-7303; MJ, Methanocaldococcus jannaschii DSM 2661; Metin, Methanocaldococcus infernus ME; Metvu, Methanocaldococcus vulcanius M7; Mefer, Methanocaldococcus fervens AG86; MK, Methanopyrus kandleri AV19; MTH, Methanothermobacter thermautotrophicus ΔH; MTM, Methanothermobacter marburgensis strain Marburg; Maeo, Methanococcus aeolicus Nankai-3; Mfer, Methanothermus fervidus DSM 2088; Rxyl, Rubrobacter xylanophilus DSM 9941; Tter, Thermobaculum terrenum ATCC BAA-798; Htur, Haloterrigena turkmenica DSM 5511; Hbor, Halogeometricum borinquense DSM 11551; Huta, Halorhabdus utahensis DSM 12940; AF, Archaeoglobus fulgidus DSM 4304; MM, Methanosarcina mazei Gö1; MA, Methanosarcina acetivorans C2A; MFS40622, *Methanocaldococcus* sp. FS406-22; MMP, Methanococcus maripaludis S2; P06130, accession number for Methanobacterium formicicum FdhB; MSM, Methanobrevibacter smithii ATCC 35061; Mvol, Methanococcus voltae A3; ANME, anaerobic methanotrophic archaea.

ANME2c-FsrII-6D-N and *Mj*FsrI-N are homologs of the F_420_H_2_ dehydrogenase subunit F of F_420_H_2_:quinone oxidoreductase (FqoF) complex of *A. fulgidus* (32) and F_420_H_2_:phenazine oxidoreductase (FpoF) complex of Methanosarcina mazei (Fig. 4) (19, 22, 33). Both *A. fulgidus* FqoF and *M. mazei* FpoF carry two CX_2_CX_2_CX_3_CP or ferredoxin-type [Fe_4_-S_4_] cluster motifs (Fig. 4, motifs A and B). While the motif A features are conserved in all FsrI-Ns and FsrII-Ns, motif B of FsrI and FsrII lacked the second position Cys, and this position was occupied by a His in *Mj*FsrI-N and Gln in ANME2c-FsrII-6D-N (Fig. 4). In addition, in FsrII, the terminal Pro residue was absent (Fig. 4). We also found that FsrI, FsrII, FpoF, and FqoF carry four more conserved cysteine residues, and we called this set motif C (Fig. 4). Since a solved three-dimensional structure of either an FsrI or FsrII is not available, to further gauge the potentials of iron-sulfur cluster assembly by cysteine residues of motifs A, B, and C in ANME2c-FsrII-6D-N and *Mj*FsrI-N, we developed *in silico* models for these proteins using AlphaFold2 (34, 35).

**FIG 5:**
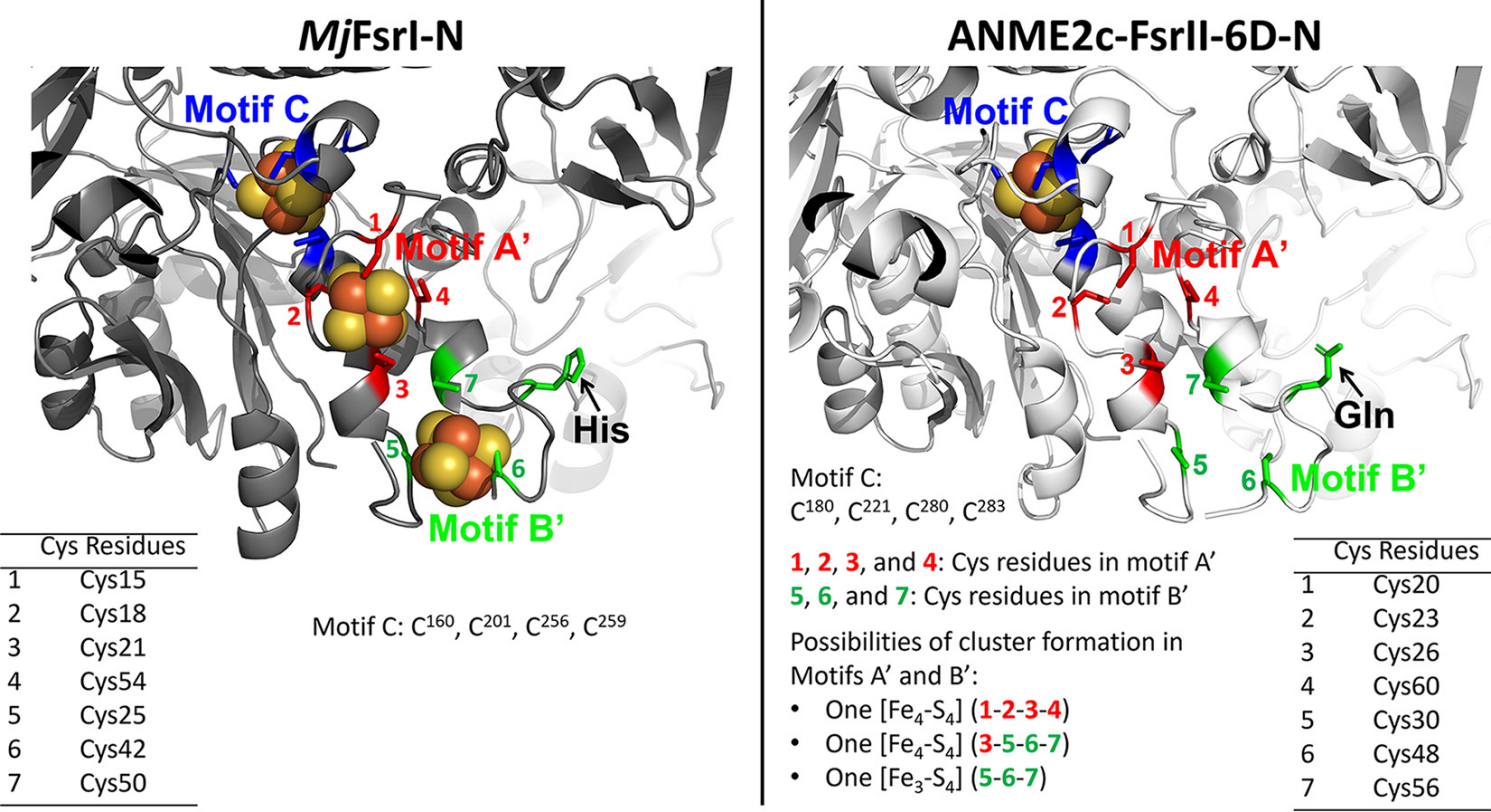
Iron-sulfur cluster binding residues of *Mj*FsrI-N and ANME2c-FsrII-6D-N based on AlphaFold2 model prediction. Modeled structures of *Mj*FsrI-N and ANME2c-FsrII-6D-N with docked [Fe_4_-S_4_] clusters from *M. marburgensis* F_420_-reducing [NiFe]-hydrogenase (Frh; PDB ID 3ZFS) (89). Cysteine residues of motifs A′, B′, and C are shown as described for Fig. 4.

The *in silico* models presented clear possibilities for the formation of iron-sulfur clusters by motifs A and B, as well as a previously unrecognized motif C, in *Mj*FsrI-N, FpoF, and FqoF (Fig. 4 and 5 and Fig. S2). The residues involved in the structures of motif A and B were also a bit different from those identified in sequence alignment, and we called these redefined motifs A′ and B′ (Fig. 4 and 5 and Fig. S2). The AlphaFold2 model of ANME2c-FsrII-6D-N suggested that motif C could form an [Fe_4_-S_4_] cluster. However, the following structural elements bring additional potentials for cluster formation (Fig. 5): motif A′, [Fe_4_-S_4_] cluster; a combination of Cys residues 3, 5, 6, and 7, [Fe_4_-S_4_] cluster; motif B′, [Fe_3_-S_4_] cluster. Since the iron and acid labile sulfur content data predict that ANME2c-FsrII-6D carries four [Fe_4_-S_4_] clusters and ANME2c-FsrII-6D-C is predicted to hold 2 such units, from the above-mentioned modeling results we hypothesize that ANME2c-FsrII-6D-N assembles two [Fe_4_-S_4_] clusters, one of which would be in motif C and another via one of the three additional possibilities presented above.

### C-terminal domain.

**FIG 6:**
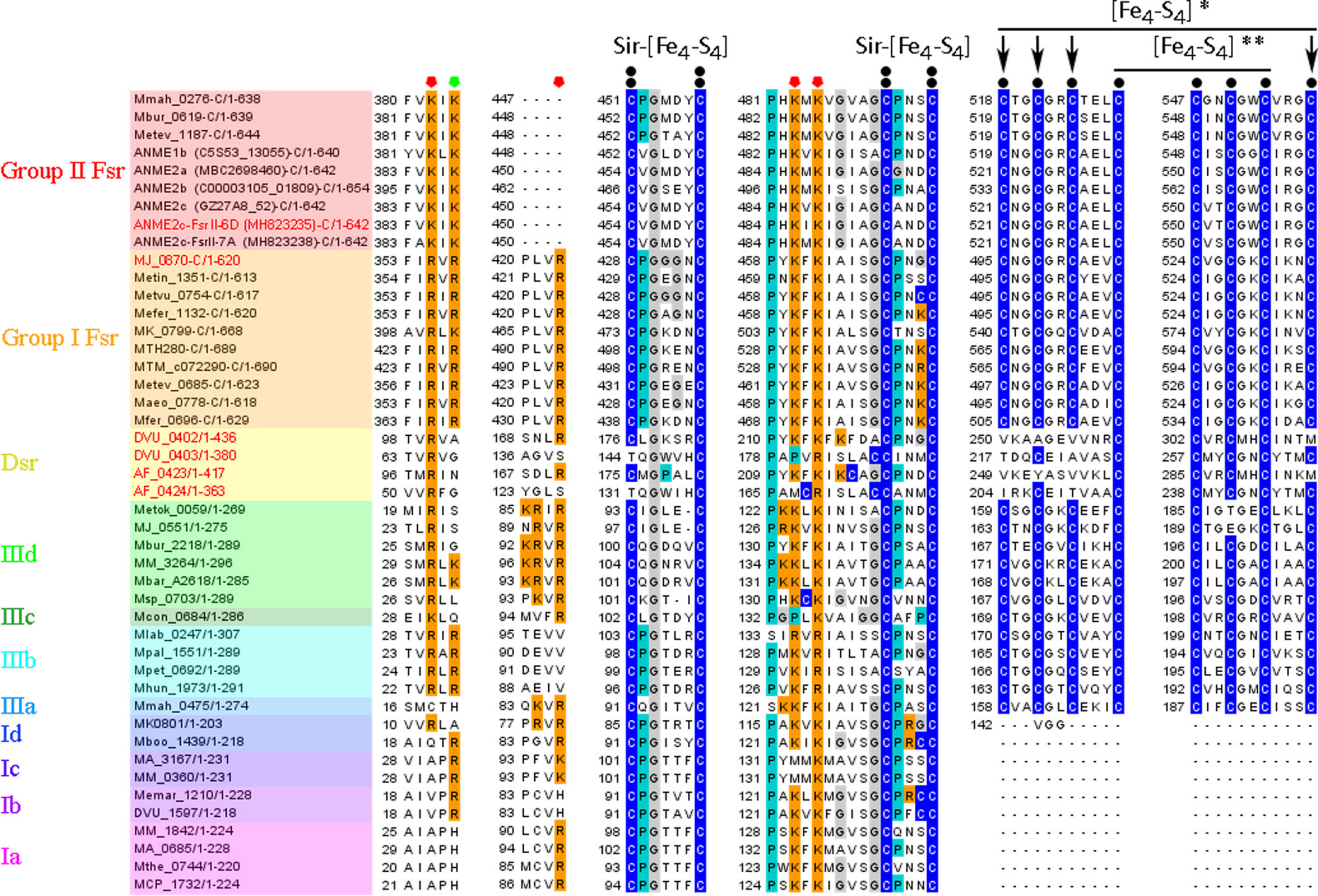
Multiple-sequence alignment of ANME2c-FsrII-6D-C homologs. Amino acid residues are represented as follows: blue, cysteine; cyan, proline; gray, glycine; orange, positively charged (H, R, or K). Black circles represent conserved cysteine residues, double black bullets represent siroheme-[Fe_4_-S_4_] cluster binding residues, red polygons show sulfite binding residues, the green polygon shows conserved positively charged residues [RK] in group I and group II Fsrs, black bullets with overlines show [Fe_4_-S_4_] cluster assembly motifs, and single and double asterisks show peripheral and additional [Fe_4_-S_4_] centers, respectively (22). ORF numbers of the proteins are presented as described in the legend to Fig. 4. “-C” indicates that only the C-terminal part of the polypeptide is shown. Range following slashes are amino acid residues for the complete protein. Dsr, dissimilatory sulfite reductase; Ia-d, group I dissimilatory sulfite reductase like protein (Dsr-LP) subgroups a to d; IIIa-d, group III Dsr-LP subgroups a to d. The names of proteins that are of particular interest in the study are shown in red. ANME2c-FsrII-6D-C, MH823235; *Mj*FsrI-C, MJ_0870; Dv-DsrA/B, Dsr subunits A and B of Desulfovibrio vulgaris strain Hildenborough, DVU_0402 and DVU_0403; Af-DsrA/B, Dsr subunits A and B of Archaeoglobus fulgidus DSM 4304, AF_0423 and AF_0424. Metok, Methanothermococcus okinawensis IH1; Mbar, Methanosarcina barkeri strain Fusaro; Msp, Methanosphaera stadtmanae DSM 3091; Mcon, Methanosaeta concilii GP-6; Mlab, Methanocorpusculum labreanum Z; Mpal, Methanosphaerula palustris E1-9c; Mpet, Methanoplanus petrolearius DSM 11571; Mhun, Methanospirillum hungatei JF-1; Mboo, *Candidatus* Methanoregula boonei 6A8; Memar, Methanoculleus marisnigri JR1; Mthe, Methanosaeta thermophila PT; MCP, Methanocella paludicola SANAE. The legend to Fig. 4 presents abbreviations of other organisms’ names.

**FIG 7:**
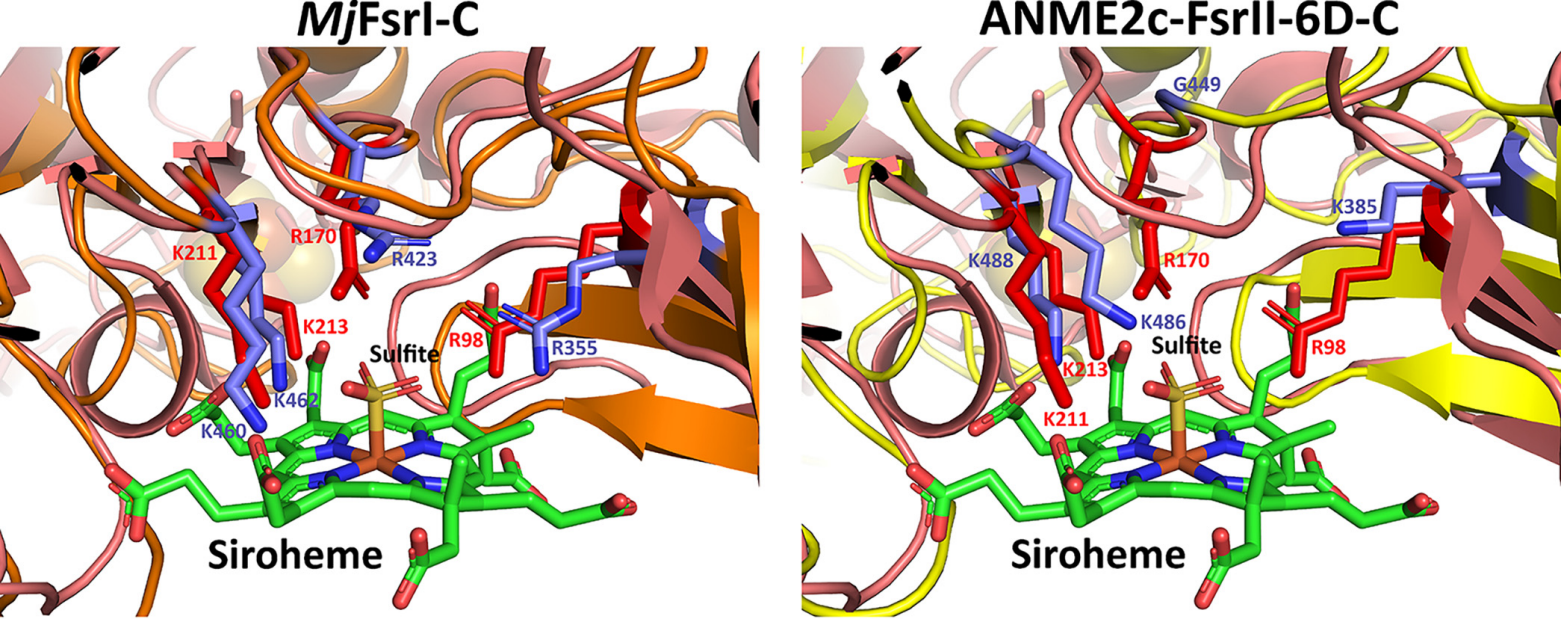
Sulfite binding pocket of *Mj*FsrI-C and ANME2c-FsrII-6D-C. Structural alignments of *A. fulgidus* DsrA with *Mj*FsrI-C and ANME2c-FsrII-6D-C, respectively. The protein backbones of *A. fulgidus* DsrA, *Mj*FsrI-C, and ANME2c-FsrII-6D-C are in deep salmon, orange, and yellow, respectively. Carbon atoms of residues involved in the formation of sulfite binding site of *A. fulgidus* DsrA and *Mj*FsrI-C/ANME2c-FsrII-6D-C are in red and purple, respectively. The residue numbers correspond to *A. fulgidus* DsrA (red) and *Mj*FsrI-C/ANME2c-FsrII-6D-C (purple). For siroheme and sulfite the following color code is used: green, carbon; blue, nitrogen; red, oxygen; yellow, sulfur; orange, iron.

The C-terminal half of ANME2c-FsrII-6D (ANME2c-FsrII-6D-C) carried sequence features that are typical of a siroheme-[Fe_4_-S_4_] cluster and two additional [Fe_4_-S_4_] clusters of *Mj*FsrI-C and DsrA, but, as elaborated below, it differed from the latter two in terms of sulfite binding residues (Fig. 6 and 7).

### (i) Peripheral and additional [Fe_4_-S_4_] clusters.

**FIG 8:**
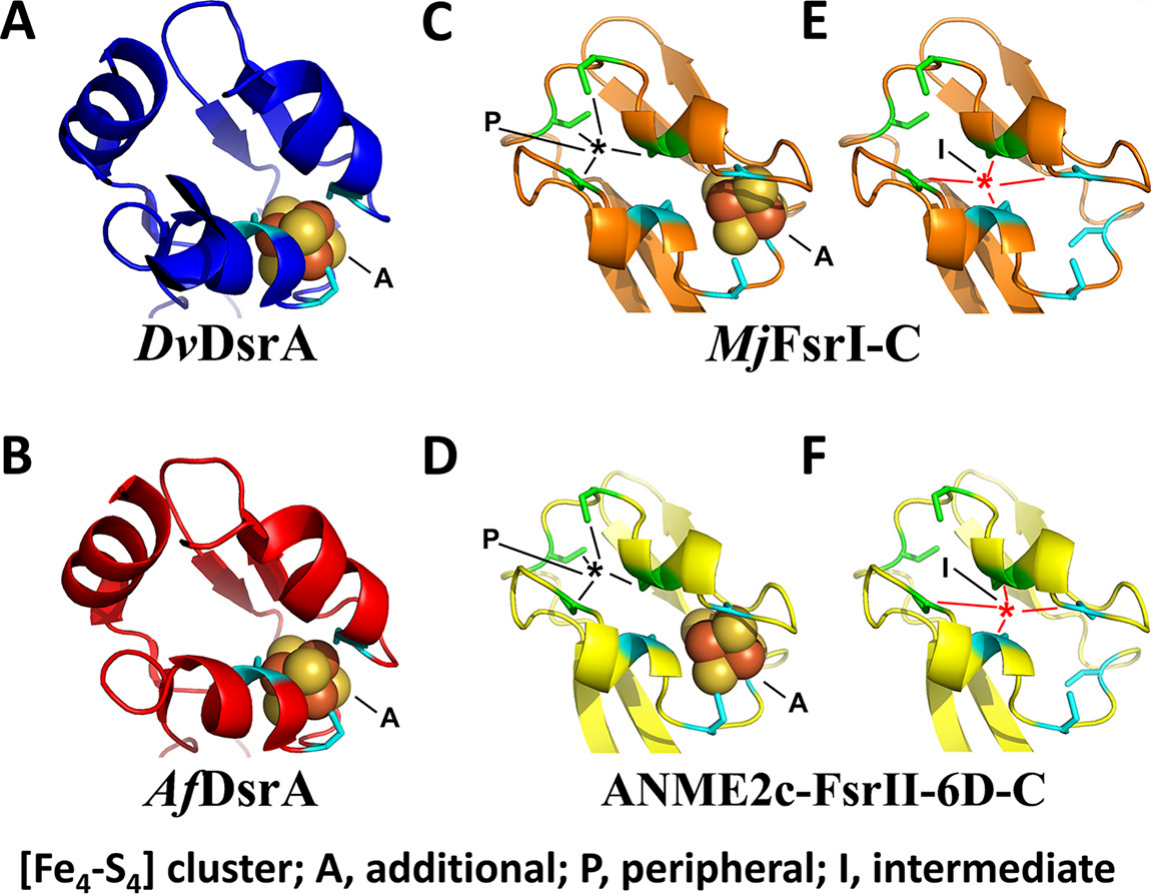
[Fe_4_-S_4_] cluster binding pockets of Fsr-C homologs. (A) Desulfovibrio vulgaris DsrA (*Dv*DsrA; PDB ID 2V4J). (B) Archaeoglobus fulgidus DsrA (*Af*DsrA; PDB ID 3MM5). (C and E) *Mj*FsrI-C. (D and F) ANME2c-FsrII-6D-C. Carbon atoms of conserved cysteine residues involved in the binding of a [Fe_4_-S_4_] cluster are colored as follows: DsrA (as in panels A and B), cyan; additional and peripheral [Fe_4_-S_4_] clusters of *Mj*FsrI-C and FsrII-C (as in panels C to F), cyan and green, respectively.

In the current study, an AlphaFold2 modeling and alignment with X-ray crystallographic structures of *A. fulgidus* and *D. vulgaris* DsrA subunits (PDB IDs 3MM5 and 2V4J) suggested that a set of Cys residues in a configuration that is different from what was previously proposed (19, 22) contribute to peripheral and additional [Fe_4_-S_4_] clusters in all FsrI-Cs and FsrII-Cs (Fig. 6 and 8). These newly recognized motifs were CX_2_CX_2_CX_n_C (peripheral) and CX_n_CX_2_CX_2_C (additional) (Fig. 6). Of these, the peripheral [Fe_4_-S_4_] cluster is not present in *A. fulgidus* and *D. vulgaris* DsrA or DsrB subunits (Fig. 6). In *A. fulgidus* and *D. vulgaris* DsrAs, which are the catalytic subunits, the space required for the binding of the peripheral [Fe_4_-S_4_] cluster is present, but it lacks cysteine residues (Fig. 8A and B). The relative locations of the Cys residues for peripheral and additional clusters in FsrI-C and FsrII-C have brought up three possibilities for the [Fe_4_-S_4_] cluster formation in these units (Fig. 8): (i) assembly two of [Fe_4_-S_4_] clusters, peripheral and additional (Fig. 8C and D); (ii) only the additional cluster as seen in DsrA (Fig. 8C and D); (iii) only one [Fe_4_-S_4_] cluster positioned in the middle of the peripheral and additional site and formed by utilizing two Cys residues from each site (Fig. 8E and F). The [Fe_4_-S_4_] clusters representing these possibilities will experience different protein environments, and therefore, will exhibit different redox properties and midpoint redox potential values (E^0^′). In summary, both FsrI-C and FsrII-C are predicted to hold two to three [Fe_4_-S_4_] clusters, one of which may be ligated to the siroheme, whereas DsrAs in *A. fulgidus* and *D. vulgaris* assemble only one [Fe_4_-S_4_] cluster in addition to the siroheme-[Fe_4_-S_4_] unit.

### (ii) Sulfite and nitrite binding sites.

The sulfite binding site of a DsrA subunit of a dissimilatory sulfite reductase is comprised of four positively charged residues (PDB IDs 3MM5 and 2V4J) (36, 37). In *A. fulgidus* DsrA, these residues are R^98^, R^170^, K^211^, and K^213^ (Fig. 6) and the same residues also coordinate the nitrogen and oxygen atoms of nitrite (27). An analysis of the respective primary structures suggested a similar situation exists in FsrI-Cs, while some distinctions were observed for FsrII-Cs (Fig. 6 and 7). Specifically, FsrII-C lacked the second of the four sulfite binding residues (Fig. 6) and structural modeling suggested that in ANME2c-FsrII-6D-C this Arg residue was substituted with a Gly (Fig. 7) (14). All Arg residues of the sulfite binding site of ANME2c-FsrII-6D-C were also replaced with Lys residues (Fig. 6 and 7). These replacements reduce the side chain length, and glycine does not have a charged side chain and is smaller than Arg. Thus, the above-mentioned changes created a larger and less positively charged pocket at the sulfite binding site in ANME2c-FsrII-6D-C than that in *Mj*FsrI. These features likely provide an enhanced structural flexibility in the positioning of the substrate and perhaps providing the enzyme an ability to bind other substrates that are larger than sulfite (14) (Fig. 7). However, similar to *Mj*FsrI, ANME2c-FsrII-6D was unable to reduce thiosulfate which is larger than sulfite. It remains to be determined whether ANME2c-FsrII-6D reduces a larger substrate with a sulfonate group. The above-mentioned structural departures were not responsible for a lack of sulfite reduction by the ANME2c-FsrII-6D with F_420_H_2_ because when reduced methyl viologen (MV^·+^) was used as an electron donor, the enzyme was able to reduce this oxyanion (Fig. 3D and E).

### Iron-sulfur cluster contents of ANME2c-FsrII-6D and *Mj*FsrI.

For the combined N-terminal and C-terminal domains the *in silico* model yielded the following possibilities in terms of the nature and number of the iron-sulfur cluster: *Mj*FsrI, 5 or 6 [Fe_4_-S_4_] clusters; ANME2c-FsrII-6D, 4 or 5 [Fe_4_-S_4_] clusters with or without an [Fe_3_-S_4_] cluster. Our chemical assays suggested that ANME2c-FsrII-6D carries 15 Fe and 15 acid-labile sulfur units which closely correspond to four [Fe_4_-S_4_] clusters. The difference between the iron-sulfur cluster contents of *Mj*FsrI and ANME2c-FsrII-6D likely would pertain to the N-terminal domain or specifically the motif B of this unit.

## DISCUSSION

This study began with the hypothesis that the catalytic features of FsrII and its ecophysiological roles are distinct from those of FsrI and that this specialization likely has a substantial effect on AOM (14), a globally important geochemical process (1). The significance of these possibilities called for an examination of the catalytic properties of the FsrII. Accordingly, we generated a recombinant form of the FsrII from ANME-2c archaea in Methanosarcina acetivorans, and purified the enzyme, and characterized its biochemical properties. This is the first case where a homogenous and active form of an ANME enzyme with elaborate metallocenters was produced using a methanogen as the expression host, purified, and biochemically characterized. An ANME methyl-coenzyme M reductase, also a metalloenzyme, was previously expressed in *M. acetivorans*; however, the recombinant enzyme has yet to be purified to homogeneity (38). We discuss below our finding that the ANME-2c FsrII is an F_420_H_2_-dependent nitrite reductase, the structural basis for this specialization, and its possible ecophysiological relevance.

### Generation of ANME-FsrII in a recombinant and homogeneous form with predicted prosthetic groups and enzymatic activity.

The methanotrophic ANME-2c archaea have yet to be isolated in pure culture, and the generation of a significant amount of biomass for ANME-2c consortia, with a doubling time of several weeks, is challenging (39, 40). Therefore, the use of a recombinant system for expressing and characterizing ANME proteins offers a method for enhancing our understanding of ANME physiology and protein function in the absence of pure cultures of these archaea. Prior work has shown that a careful selection of a phylogenetically related genetically tractable host matters in both successful heterologous expression of an Fsr and achieving activity in the recombinant protein (20). For example, while E. coli is a commonly used host for heterologous expression of proteins, it is unsuitable for the expression of *Mj*FsrI in a soluble and active form. On the other hand, in Methanococcus maripaludis, a close relative of *M. jannaschii* (41), *Mj*FsrI could be overexpressed with activity (20). We initially attempted to express ANME2c-FsrII-6D using *M. maripaludis* as the host; however, a mass-spectrometric analysis of cell extracts using a previously described method (42) showed that the produced protein was barely above detection in the recombinant strain (data not shown). Additionally, in assays with either F_420_H_2_ or reduced methyl viologen (MV^·+^) as the electron donor, the cell extracts did not exhibit a sulfite reductase activity (data not shown). All follow-up work with ANME2c-FsrII-6D was subsequently conducted in Methanosarcina acetivorans as the expression host, which presented several advantages over *M. maripaludis* for this study. Not only is *M. acetivorans* a genetically amenable methanogen, but this archaeon is also phylogenetically closely related to the ANME-2c, both belonging to the order *Methanosarcinales* (2, 6, 43). This relatedness provided an enhanced possibility of proper folding for the heterologously expressed ANME2c-FsrII-6D. Another important consideration is that *M. acetivorans* increased the likelihood of assembling a suitable type of siroheme in the recombinant ANME-2c protein. The siroheme structures are known to carry organism-specific modifications, such as the amidation of one of the acetate chains in Desulfovibrio desulfuricans and Allochromatium vinosum (44, 45). *M. acetivorans* lacks Fsr but carries four homologs of dissimilatory sulfite reductase-like proteins (Dsr-LPs) (22), and these Dsr-LPs have the structural features for assembling siroheme-[Fe_4_-S_4_] clusters (22), and a representative of this protein (P_590_) carrying a siroheme unit has been isolated from a *Methanosarcina* species (46). As mentioned above, *M. maripaludis*, which lacks Fsr and possesses a Dsr-LP (20, 22), can generate active *Mj*FsrI from a cloned gene (20). Consequently, the selection of *M. acetivorans* increased the probability of proper folding for the heterologously expressed ANME FsrII protein and the incorporation of a proper type of siroheme into it. Indeed, *M. acetivorans* carrying an expression vector that we constructed in this study, pDS701, produced recombinant ANME-FsrII with activity and a UV-visible spectrum that is characteristic of a siroheme protein (Fig. 2A and 3). This result also confirmed that *M. acetivorans* can produce siroheme.

### ANME2c-FsrII-6D, F_420_H_2_-dependent nitrite reductase (FNiR) with an F_420_H_2_-nonutilizing sulfite reduction activity.

The FsrII of ANME-2c is a structural homolog of *Mj*Fsr-I which reduces sulfite to sulfide with F_420_H_2_ as a reductant (19), and it was unexpected to find that this FsrII could not reduce sulfite using F_420_H_2_ as the electron donor. Since nearly all characterized sulfite reductases also reduce nitrite (19, 26–29, 47) (Table S1) and *Mj*FsrI exhibits this activity (E. F. Johnson, C. Heryakusuma, and B. Mukhopadhyay, unpublished data), we examined the potential for ANME2c-FsrII-6D to reduce nitrite. Assays with the ANME-2c FsrII confirmed active reduction of nitrite to ammonia with F_420_H_2_ as the reductant, with a *K_m_* value for nitrite (5 μM) that is comparable to that of bona fide nitrite reductases described from other organisms (Table S1). The *K_m_* value of ANME2c-FsrII-6D for the electron donor F_420_H_2_ was also comparable to that of other F_420_H_2_-utilizing enzymes, including *Mj*FsrI and F_420_H_2_-dependent thioredoxin reductase (DFTR) from *M. jannaschii*, FpoF of Methanosarcina mazei, and FqoF of Archaeoglobus fulgidus (Table S1) (19, 32, 33, 48, 49). Therefore, the F_420_H_2_-dependent nitrite reductase activity of ANME2c-FsrII-6D appears to be physiologically relevant.

A comparison of the respective kinetic constant values (Table 1) revealed valuable mechanistic information for the nitrite, hydroxylamine, and sulfite reduction activities. The apparent *K_m_* value for sulfite was 26 times higher than that of nitrite, and this property was consistent with ANME2c-FsrII-6D being a nitrite reductase. The catalytic efficiency (*k*_cat_/*K_m_*) values for nitrite and sulfite reduction could not be compared, as these two reactions had to be measured with different electron donors (Table 1), although individually, they provided key insights. The reduction of nitrite to ammonia by nitrite reductases generally proceeds through intermediate formation of hydroxylamine, and nitrite reductases frequently exhibit hydroxylamine reductase activity (26–31). Consistent with this, ANME2c-FsrII-6D exhibited an F_420_H_2_-dependent hydroxylamine reductase activity with a physiologically relevant *K_m_* value for NH_2_OH (Table 1 and Table S1). The catalytic efficiency of this activity was 3-fold higher than that for the overall nitrite reduction reaction, suggesting that this enzyme was capable of preventing the intracellular accumulation of hydroxylamine, which is toxic to many organisms (50). Based on our characterization of nitrite and hydroxylamine activity from ANME2c-FsrII-6D, it appears that the function of the FsrII clade in ANME-2 (14) is distinct from that of FsrI in the hyperthermophilic methanogen *M. jannaschii*, with the ANME-2c version of this enzyme serving as a nitrite, rather than sulfite reductase, utilizing F_420_H_2_ as a reductant. We propose naming this enzyme F_420_H_2_-dependent nitrite reductase (FNiR).

### Basis for discrimination between nitrite and sulfite in ANME2c-FsrII-6D.

Our initial analyses examined the ability of the Fsr enzyme to recognize its predicted substrate, which was sulfite. The potential oxyanion binding site of ANME2c-FsrII-6D differed structurally from those of dissimilatory sulfite reductases or Dsrs to some extent (Fig. 7), and the apparent *K_m_* of the enzyme for sulfite (~130 μM) was 11-fold higher than that of *Mj*FsrI (12 μM) (19), suggesting that sulfite may not be the preferred substrate for this enzyme. Also, sulfite is a poor competitive inhibitor of nitrite reduction by ANME2c-FsrII-6D, as the *K_i_* for sulfite was about 26 times higher than the *K_m_* for nitrite (Fig. 3B and G). However, as discussed below, these factors did not underlie the observed lack of F_420_H_2_-dependent sulfite reductase in ANME2c-FsrII-6D. The conclusion for the other substrate, F_420_H_2_, was similar, as the enzyme exhibited both physical interaction with F_420_ and catalytic activity with F_420_H_2_, even though its putative F_420_-binding site deviated structurally from that of *Mj*FsrI and Methanothermobacter marburgensis FrhB; ANME2c-FsrII-6D bound to the F_420_ affinity column and exhibited an apparent *K_m_* for F_420_H_2_ (~14 μM) that was comparable to that of *Mj*FsrI (21 μM) (19) (Table S1). Thus, the absence of F_420_H_2_-dependent sulfite reductase activity in ANME2c-FsrII-6D was unlikely to be due to a lack of substrate recognition by the enzyme.

The overall thermodynamics of the redox reaction between F_420_H_2_ and the oxyanions was not a possible factor contributing to the unexpected activity profile for ANME2c-FsrII-6D, as the respective Δ*G*°′ values predict that the reduction of both sulfite and nitrite with F_420_H_2_ are thermodynamically feasible (equations 4 and 5). The enzyme reduced nitrite, hydroxylamine, and MV^·+^ but not sulfite with F_420_H_2_. However, with MV^·+^ as the electron donor, ANME2c-FsrII-6D exhibited a robust sulfite reductase activity with high *k*_cat_ and catalytic efficiency (*k*_cat_/*K_m_*) (Table 1). These suggested that in ANME, the reducing equivalents derived from F_420_H_2_ were not suitable for reducing sulfite.

(1) $${\text{F}}_{\text{42}0}+{\text{ 2e}}^{-}+{\text{2H}}^{+}\to {\text{ F}}_{\text{42}0}{\text{H}}_{2}\;\;\;\;{\text{E}}^{0}\text{′}=\;-\text{36}0\text{ mV}$$

(2) $${\text{NO}}_{\text{2}}{}^{-}+{\text{ 6e}}^{-}+{\text{ 8H}}^{+}\to {\text{ NH}}_{\text{4}}{}^{+}+{\text{ 2H}}_{2}\text{O}\;{\text{E}}^{0}\text{′}=\;+\text{44}0\text{ mV}$$

(3) $${\text{HSO}}_{\text{3}}{}^{-}+{\text{ 6e}}^{-}+{\text{ 6H}}^{+}\to {\text{ HS}}^{-}+{\text{ 3H}}_{\text{2}}\text{O}\;{\text{E}}^{0}\text{′}=\;-\text{116 mV}$$

(4) $${\text{NO}}_{\text{2}}{}^{-}+{\text{ 3F}}_{\text{42}0}{\text{H}}_{2}+{\text{ H}}^{+}\to {\text{ NH}}_{\text{4}}{}^{+}+{\text{ 2H}}_{\text{2}}\text{O }+{\text{ 3F}}_{\text{42}0}\Delta G^{\circ }\text{′}=\;-\text{457 kJ}/\text{mol}$$

(5) $${\text{HSO}}_{\text{3}}{}^{-}+{\text{ 3F}}_{\text{42}0}{\text{H}}_{2}\to {\text{ HS}}^{-}+{\text{ 3H}}_{\text{2}}\text{O }+{\text{ 3F}}_{\text{42}0}\Delta G^{\circ }\text{′}=\;-\text{135 kJ}/\text{mol}$$

(6) $${\text{MV}}^{\text{2}+}+{\text{ e}}^{-}\to {\text{MV}}^{•+}{\text{E}}^{0}\text{′}=\;-\text{446 mV}$$

(7) $${\text{HSO}}_{\text{3}}{}^{-}+{\text{ 6MV}}^{•+}+{\text{6H}}^{+}\to {\text{ HS}}^{-}+{\text{ 3H}}_{\text{2}}\text{O }+{\text{ 6MV}}^{\text{2}+}\Delta G^{\circ }\text{′}=\;-\text{191 kJ}/\text{mol}$$

**FIG 9:**
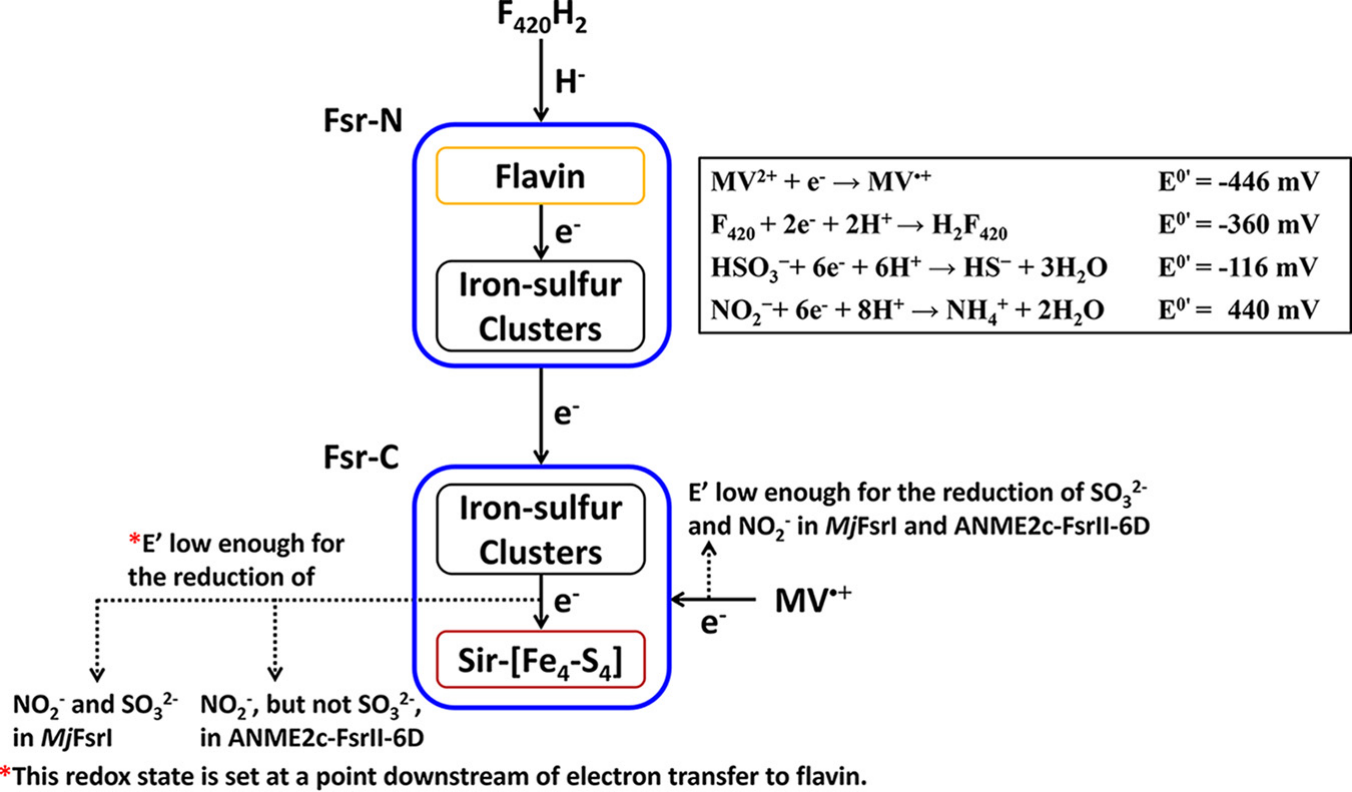
Electron transfer in F_420_-dependent sulfite reductase (Fsr). In Fsr, protein-bound flavin is reduced by F_420_H_2_, and the electrons from reduced flavin are transferred via a set of iron-sulfur clusters to the siroheme-[Fe_4_-S_4_] center, where an oxyanion is reduced. “Fsr-N” and “Fsr-C” indicate the N- and C-terminal halves of the Fsr, respectively.

Since F_420_H_2_ is a hydride donor, the first step of electron transfer in an Fsr involves FAD, the only available hydride acceptor in the enzyme residing in the N-terminal domain (Fig. 9). Then, these electrons are transported through the iron-sulfur cluster systems of the N- and C-terminal domains to the siroheme-[Fe_4_-S_4_] center at Fsr-C, where an oxyanion is reduced (19, 22). It is possible that one or more of these intermediate electron carriers, or even the one donating directly to the oxyanion reduction site, operate at a redox potential that is higher than that of the primary reductant, F_420_H_2_, thereby raising the redox potential of the retrieved electrons and making these less potent reductants. On the other hand, MV^·+^ likely transfers the electrons directly to the siroheme-[Fe_4_-S_4_] center, thus bypassing the redox potential-altering steps. Accordingly, we hypothesize that although both F_420_H_2_ (E^0^′, −360 mV; equation 1) and MV^·+^ (E^0^′, −446 mV; equation 6) are thermodynamically competent in reducing sulfite (E^0^′, −116 mV; equation 3) and nitrite (E^0^′, +440 mV; equation 2), in ANME2c-FsrII-6D, the electrons derived from F_420_H_2_ are delivered to the siroheme-site at a redox potential value that is too high for the reduction of sulfite and yet suitable for nitrite reduction. In contrast, the electron transfer path in *Mj*FsrI retains the reducing power of electrons derived from F_420_H_2_ to a level that is suitable for reducing sulfite, which would obviously favor nitrite reduction, an easier task. Since MV^·+^ can deliver electrons to the siroheme-[Fe_4_-S_4_] center without such an alteration, it facilitates the reduction of both oxyanions for both *Mj*FsrI and ANME2c-FsrII-6D. These results suggest that the siroheme-[Fe_4_-S_4_] centers of these two enzymes are equally competent in oxyanion reduction.

We rationalize the above-stated hypothesis in terms of the cofactor contents and modeled structures of ANME2c-FsrII-6D and *Mj*FsrI (Fig. 5 and 7). The siroheme and flavin contents of ANME2c-FsrII-6D were consistent with the expectations from the known properties of *Mj*FsrI, FpoF, and FqoF (19, 32, 48), and a similar agreement was seen for the predicted numbers and natures of the C-terminal iron-sulfur clusters. However, ANME2c-FsrII-6D-N seemingly differed from *Mj*FsrI-N in terms of the iron-sulfur cluster contents (Fig. 4 and 5), predicting distinct redox properties for these units. Also, a major change in the charge environment (from positive to negative) at the F_420_-binding site of ANME2c-FsrII-6D as predicted from a primary structure comparison could impact the electron transfer process. For these distinctions, the FsrII of ANME-2c (ANME2c-FsrII-6D) and FsrI of *M. jannaschii* (*Mj*FsrI) could deliver F_420_H_2_-derived electrons to the siroheme-[Fe_4_-S_4_] center at different redox potentials.

### Potential ecophysiological role of F_420_H_2_-dependent nitrite reductase in ANME.

The FsrII homologs occur in ANME and in selected psychrophilic and mesophilic methanogens from marine and hypersaline habitats, all belonging to the phylum *Halobacteriota* (6, 14, 19, 22). In contrast, as mentioned above, FsrI occurs exclusively in certain methanogens that belong to the phylum *Methanobacteriota*, with widespread presence in vent isolates, sporadic occurrence in thermophiles from hot springs, sewage digesters, and salt lagoons, and rare existence in mesophilic species (6, 14, 19, 22). The only exception to this distribution is Methanohalobium evestigatum, a moderate thermophile from *Halobacteriota* that was isolated from a salt lagoon, as it appears to carry both FsrI and FsrII (14). These distinctions in the distribution of FsrI and FsrII bring up the possibility that these clades may be optimized for the different environments and host physiology.

The initial presumption of FsrII having a role in sulfite detoxification was reasonable considering the highly reducing methane seep sediments, where elevated concentrations of sulfide and sulfur intermediates from sulfate-coupled AOM are common (16, 51, 52) and where transient oxygen exposure from bioturbation or methane ebullition could generate sulfite (19, 22). A similar situation has been suggested for the deep-sea hydrothermal vent habitat of *M. jannaschii* (19, 22). Sulfite is known to inactivate methyl coenzyme M reductase (Mcr) in methanogens, including members of the *Methanosarcinales* (53, 54), and *Mj*FsrI protects *M. jannaschii* from this damage (19, 20). As Mcr is essential in methanogens and ANME, catalyzing both the last step of methanogenesis and the first step of ANME methanotrophy (12, 43), it is reasonable to assume that ANME archaea are also likely similarly sensitive to sulfite. Indeed, methane seep sediment microcosm experiments using ANME-2c as a dominant methane-oxidizing archaeon showed that sulfite inhibited rates of methane oxidation (14). It was therefore surprising that ANME2c-FsrII-6D appeared incapable of reducing sulfite with its natural electron carrier F_420_.

The demonstration of F_420_H_2_-dependent nitrite reductase activity by ANME2c-FsrII-6D introduces new ideas about the potential ecophysiological role of FsrII. Even at low micromolar concentrations, nitrite has been shown to oxidize the Ni(I) center of F_430_ in Mcr in methanogens, rendering this essential enzyme inactive (55, 56). This observation leads to a hypothesis that FNiR is a nitrite detoxification enzyme for ANME-2c, playing a physiological role that is analogous to that of *Mj*FsrI with respect to sulfite (19, 20). *M. jannaschii* overexpresses FsrI in response to the presence of sulfite in the growth medium (19, 20), and deletion of the *fsr* gene results in sulfite sensitivity in this methanogen (21). An ANME FsrII has been found to be overexpressed under *in situ* conditions; however, corresponding information on porewater nitrogen concentrations and speciation was unfortunately not provided in these studies (14–16, 24).

Nitrite has been measured at low concentrations within methane seeps (57), and at least one study has reported the presence of anammox (anaerobic ammonium-oxidizing) bacteria in seep sediments, which carry out a redox process that couples ammonia oxidization with nitrite reduction (58), suggesting that exposure of ANME to nitrite in marine seeps is possible. The involvement of FsrII in nitrite detoxification is also interesting from the perspective of its phylogenetic distribution, as it appears to be common among marine ANME lineages but conspicuously lacking in ANME-2d (*Candidatus ‘Methanoperedens nitroreducens’*), a close methanotrophic relative found in terrestrial environments that is capable of directly oxidizing methane with nitrate reduction (59). In this ANME lineage, nitrite is predicted to be oxidized by a nitrite reductase (*nrfAH*) using menaquinol as the electron donor (60). These biochemical findings and structural predictions point to new research directions for increasing our understanding of the ecophysiological role of FsrII in ANME and its evolutionary history.

In summary, through heterologous expression in a related host methanogen, this study uncovered novel functionality of the group II Fsr in uncultured methanotrophic ANME-2 archaea. In ANME-2, Fsr group II appears to function solely as a coenzyme F_420_ utilizing nitrite reductase (FNiR), a role that is notably distinct from group I Fsr. In the hyperthermophilic methanogen *M. jannaschii*, FsrI catalyzes F_420_ coupled sulfite reduction and may also serve as a nitrite reductase. The FsrII enzyme is hypothesized to restrict the use of sulfite via fine-tuned control of the redox potential of the electrons delivered at its oxyanion reduction site. If the prediction about the physiological role of ANME-2c FsrII is proven to be correct, it would show that both FsrI and FsrII protect the same enzyme, methyl coenzyme M reductase (Mcr), from inactivation by two structurally similar oxyanions, sulfite and nitrite, respectively, in archaea from different habitats involved in methane production and consumption. Our study also showed that subtle changes in the predicted protein structure likely alters the properties of the redox centers, contributing to functional diversification in the Fsr protein family. These insights, along with the available heterologous expression system for an ANME-FsrII (this study) and *Mj*FsrI (20) and a recently developed genetic analysis system for *M. jannaschii* (21), will allow detailed characterization of the Fsrs, which seem to be key factors in major marine geochemical processes. This genetic toolbox alongside recent methodological innovations in the structural analyses of proteins (61) shows significant potential for expanding our mechanistic understanding of redox active proteins in uncultured or difficult-to-grow archaea.

## MATERIALS AND METHODS

### Growth of Methanosarcina acetivorans.

*M. acetivorans* C2A strain WWM60 (62) was grown in a high-salt medium with 50 mM trimethylamine (HS-TMA) as a methanogenic substrate as described previously (63). For growth in liquid culture, 25 mL or 300 mL of HS-TMA medium in a 160-mL or 530-mL serum bottle (Wheaton Science Products, Millville, NJ), respectively, was used. Inoculated cultures were incubated at 37°C with shaking at 75 rpm in a C24 incubator shaker (New Brunswick Scientific, Edison, NJ). For growth on solid medium, agar (2% [wt/vol]) was added as a solidifying agent to the medium, and the inoculated plates were placed inside an Oxoid anaerobic jar (model HP0011A; Thermo Fisher Scientific, Waltham, MA) which was sealed and filled with a mixture of N_2_ and CO_2_ (80:20 [vol/vol]) containing 7.5 ppm of H_2_S at a pressure of 10^5^ Pa. The jar was left inside the anaerobic chamber (Coy Laboratory Products, Inc., Grass Lake, MI), which contained a mixture of N_2_, CO_2_, and H_2_ (76:20:4 [vol/vol/vol]), and maintained at 37°C. To select *M. acetivorans* strains harboring pDS701, an *M. acetivorans*–Escherichia coli shuttle vector, puromycin was added to the solid or liquid medium at a final concentration of 2 or 1 μg/mL, respectively.

### Construction of an M. acetivorans strain expressing ANME FsrII and expression of the heterologous protein.

The coding sequence for FsrII was previously PCR amplified from the metagenomic DNA extracted from an ANME-2-dominated methane seep sediment and cloned into the plasmid pCR4-TOPO (14). For the current study, from one such clone, called Fsr-5207-6D, an expression vector for FsrII, named pDS701, was developed by following a previously described method (62); the details of this step appear in the supplemental material. This vector was introduced into *M. acetivorans* using a liposome-mediated transformation method, and the transformant was selected on HS-TMA solid medium containing puromycin (64). The expression of the recombinant protein, called ANME2c-FsrII-6D, in *M. acetivorans*(pDS701) was induced by addition of tetracycline to the liquid culture to a final concentration of 100 μg/mL, and the induced cells were examined for the expression of the heterologous protein via SDS-PAGE analysis of cell extracts.

### Purification of ANME2c-FsrII-6D.

Heterologously expressed ANME2c-FsrII-6D was purified primarily by following a previously described procedure (19). All steps described below, except for the centrifugation of materials with volumes larger than 20 mL, occurred inside an anaerobic chamber that contained a mixture of N_2_ and H_2_ (96:4 [vol/vol]) and maintained at room temperature (~25°C). For the centrifugation of higher-volume materials, a Nalgene centrifuge bottle (MilliporeSigma, Burlington, MA) was filled and sealed inside the chamber, then centrifuged outside, and taken back into the chamber for the collection of the supernatant and pellet. All column-chromatographic steps were performed employing gravity flow. Followings are the details of the purification experiment.

Six grams of wet cell pellet of *M. acetivorans*(pDS701) containing recombinant ANME2c-FsrII-6D was resuspended in 30 mL of a 100 mM potassium phosphate buffer, pH 7 (buffer A), containing 0.2 mg/mL DNase I (MilliporeSigma, Burlington, MA). The resulting suspension that contained the lysed cells resulting from the osmotic shock in the low-ionic-strength solution was homogenized via serial passages through 18-, 22-, and 25-gauge needles in that sequence. The lysate was centrifuged at 48,000 × *g* and 4°C for 20 min. The supernatant obtained from this step was fractionated on ice by precipitation with ammonium sulfate at two sequential stages, representing 30% and 60% saturations of the salt. At each stage, the suspension was centrifuged at 48,000 × *g* and at 4°C for 20 min. The pellet from 60% (NH_4_)_2_SO_4_ saturated extract was dissolved in a 1 M (NH_4_)_2_SO_4_ solution prepared in buffer A and fractionated over chromatography resins, each packed in a 1- by 20-cm column (Econo-Column; Bio-Rad Laboratories, Inc., Hercules, CA). The first step involved a column with 6 mL of phenyl-Sepharose resin (Cytiva, Marlborough, MA) that was pre-equilibrated with 1 M (NH_4_)_2_SO_4_; all (NH_4_)_2_SO_4_ solutions were prepared in buffer A. After the sample was loaded onto it, the column bed was washed with the following (NH_4_)_2_SO_4_ solutions in buffer A at 1 M, 0.75 M, 0.5 M, 0.25 M, and 0 M in that sequence, and in each case the volume was 18 mL, except that the first was 36 mL. For each wash, fractions of 6-mL volumes were collected. Eluates from 0 M (NH_4_)_2_SO_4_ wash contained Fsr activity and were pooled. This pool was loaded onto a column packed with 6 mL of QAE-Sephadex (Cytiva, Marlborough, MA) that was pre-equilibrated with buffer A. The flowthrough from the column contained Fsr activity, and the respective fractions were pooled and then loaded onto a column with 4 mL F_420_-Sepharose resin that was pre-equilibrated with buffer A; F_420_-Sepharose was prepared as described previously (19, 65, 66). The column bed was washed with the following NaCl solutions prepared in buffer A at 0 M, 0.25 M, 0.5 M, 0.75 M, and 1 M in that sequence; for the first wash, the volume was 24 mL and for each of the rest it was 12 mL. The 0.25 M NaCl fractions contained Fsr activity and were pooled. The pooled enzyme preparation was analyzed for composition via SDS-PAGE, and the observed protein bands were characterized via mass spectrometry. The enzyme preparation was also used for enzymatic activity assays.

### SDS-PAGE, mass-spectrometric analysis, and size exclusion chromatography.

SDS-PAGE was performed according to Laemmli (67). For identifying the proteins present in an SDS-PAGE gel band, the tryptic peptides generated from an in-gel digestion with trypsin were separated by one-dimensional reversed-phase chromatography and analyzed on an LTQ Orbitrap Elite mass spectrometer (Thermo Fisher Scientific) (68, 69). The acquired tandem mass spectrometry (MS/MS) data were searched using Sipros (70) against a database which was composed of the predicted proteome of Methanosarcina acetivorans (71) and a protein sequence for ANME2c-FsrII-6D (accession number QBZ96224). Initial results were filtered with a 1% false discovery rate (FDR) threshold at the peptide level estimated by the target–decoy approach (72). A minimum of two peptides, one of them unique, was required for each identified protein. Size exclusion chromatography was performed as described previously (49, 73) and detailed in the supplemental material.

### Determination of flavin content of ANME2c-FsrII-6D.

From purified ANME2c-FsrII-6D, flavin was extracted via a previously described nondegradative method (74) with some modifications. A 100-μL solution of 63.2 μg purified protein in 100 mM potassium phosphate buffer (pH 7) was combined with a 500-μL methanol-methylene chloride mixture (9:10 [vol/vol]) and vortexed vigorously for 60 s. The mixture was then supplemented with 240 μL of 0.1 M ammonium acetate pH 6.0, vortexed vigorously for 60 s, and centrifuged at 17,000 × *g* and 4°C for 5 min. Following the centrifugation, 700 μL of the aqueous phase was filtered through a 0.45-μm membrane filter (Pall Acrodisc syringe filter; Pall Corporation, Port Washington, NY). A 100-μL aliquot of this filtered extract was resolved on a 4.6- by 250-mm Vydac analytical HPLC C_18_ column (Separation Group, Hesperia, CA) by using a previously described method (75) with the following details. The HPLC instrumentation was the same as that which we used for size exclusion chromatography and described in the supplemental material. The solvents were a solution containing 2% acetonitrile and 27.5 mM sodium acetate buffer pH 4.7 (A) and 100% acetonitrile (B), and the flow rate was 0.6 mL/min. The sample was applied under a flow of 100% A, and the elution was performed with the following gradients (percent B in A): 0%, 0 to 2 min; 0% to 2%, 2 to 6 min; 2% to 10%, 6 to 15 min; 10% to 100%, 15 to 18 min; 100% (isocratic), 18 to 21 min; and 100% to 0%, 21 to 24 min. The elution was monitored at 450 nm, and the UV-visible spectra of the eluted compounds were collected by use of the diode array detector. The standards were flavin mononucleotide (FMN) and flavin adenine dinucleotide (FAD) (Thermo Fisher Scientific, Waltham, MA). The nature of the ANME2c-FsrII-6D-bound flavin was determined from the elution time and the spectrum of the eluted compound. The amount of FAD present in an extract was estimated by use of a standard plot of peak area against micromoles of FAD applied to the column.

### Enzymatic activity assays.

The F_420_H_2_-dependent sulfite and nitrite reduction activities of ANME2c-FsrII-6D were measured spectrophotometrically under strictly anaerobic conditions using reduced F_420_ (F_420_H_2_) as the reductant and following methods described previously (19, 76). It involved the monitoring of F_420_H_2_ oxidation at 400 nm, and the reaction rate was calculated using the extinction coefficient value of 25 mM^−1^ cm^−1^ (77). For each standard assay, a 0.8-mL reaction mixture containing the following components was used: 100 mM potassium phosphate buffer (pH 7), 40 μM F_420_H_2_, 0.5 mM DTT (if desired), 500 μM sodium sulfite or nitrite, and 12 μg purified ANME2c-FsrII-6D. Reduced F_420_ (F_420_H_2_) was generated via chemical reduction of F_420_ dissolved in water with NaBH_4_ (19, 49, 65), and unreacted NaBH_4_ was titrated using HCl.

The Fsr-N-specific partial activity of ANME2c-FsrII-6D was assayed using a strategy that has been described for F_420_H_2_ dehydrogenase (65) and used with *Mj*FsrI (19). Here, with F_420_H_2_, Fsr-N reduces methyl viologen (MV^2+^) to MV^·+^ (reduced methyl viologen), and metronidazole continuously removes MV^·+^, which is a colored product of the reaction, by chemically oxidizing it to colorless MV^2+^ (65). This system allows an interference-free observation of the formation of F_420_ spectrophotometrically at 400 or 420 nm and also helps to keep the concentration of MV^2+^ constant (65). In our study, the assay was performed in a 0.8-mL reaction mixture containing 100 mM potassium phosphate buffer (pH 7), 40 μM F_420_H_2_, 2.5 μM MV^2+^, and 1.5 mM metronidazole, and the reaction was monitored at 400 nm (19). The Fsr-C-specific partial reaction, the reduction of sulfite, was followed with MV^·+^ as the electron donor. Here, the measurement was performed using the Fsr standard assay as described above except that the electron donor was 500 μM reduced methyl viologen (MV^·+^), and the progress of the reaction was monitored at 560 nm (ε_560_ for MV^·+^, 8 mM^−1^ cm^−1^ [77]); MV^·+^ was generated by reducing MV^2+^ in water with a Zn wire overnight inside an anaerobic chamber (19).

The standard assay with F_420_H_2_ as the electron donor was also used to test the ability of ANME2c-FsrII-6D to reduce thiosulfate and hydroxylamine, and here, these electron acceptors were used at a final concentration of 500 μM in place of sulfite and nitrite. Similarly, the ability of the enzyme to use NADH and NADPH in place of F_420_H_2_ as electron donors was examined with sulfite and nitrite as electron acceptors, and in each case, the concentration of the reduced coenzyme was 50 μM and the progress of the reaction was followed at 340 nm. For the kinetic studies, the concentrations of the relevant substrates were varied. Coenzyme F_420_ was purified from Methanothermobacter marburgensis (19, 76, 78).

### Assays for iron, acid-labile sulfur, sulfide, ammonia, and protein.

The iron and acid-labile sulfur contents of ANME2c-FsrII-6D were estimated via bathophenanthroline and methylene blue methods, respectively (79–81). In each case, a solution of the protein in 100 mM potassium phosphate buffer, pH 7, was used. For iron estimation, which was performed aerobically, 100 μL of the protein solution (51 μg protein) was mixed with 100 μL of 325 mM acetate buffer, pH 4.5, and 50 μL of 10% (wt/vol) ascorbic acid. This mixture was diluted with 700 μL of distilled water and incubated at 25°C for 10 min. Then, 50 μL of a 0.5% (wt/vol) solution of bathophenanthroline in the acetate buffer was added to the mixture, and the absorbance of this final solution was read at 535 nm. Ferrous ammonium sulfate, Fe(NH_4_)_2_(SO_4_)_2_, was used as the standard.

For the estimation of the acid-labile sulfur content, the first few steps occurred inside an anaerobic chamber containing a mixture of N_2_ and H_2_ (96:4 [vol/vol]), and these employed a micro-gas diffusion cell made up of a 5.6-mL, 75- by 10-mm borosilicate glass Kimax tube (Duran Wheaton Kimble, Millville, NJ) with an insert of a 30- by 3.5-mm capillary tube (Duran Wheaton Kimble, Millville, NJ) (19, 82). The annular space of the diffusion cell was filled with 100 μL of anaerobic protein solution (51 μg protein), and 20 μL of 1 M NaOH was added to the capillary. The tube was closed tightly with a No. 000 rubber stopper (EPDM rubber stopper, WidgetCo, Houston, TX). Then, 100 μL of anaerobic 1 M HCl was added to the annular space of the cell with a syringe, and the tube was incubated at 25°C for 30 min to release the acid-labile sulfur as H_2_S gas; H_2_S was trapped in the NaOH solution in the capillary. At this point, the capillary was taken out of the glass tube, and its contents were diluted with 80 μL of distilled water, pipetted out into a new glass tube, and mixed with 200 μL of a 0.5 M Zn acetate solution and 550 μL of distilled water. The tube with a suspension of ZnS precipitate was sealed with a No. 000 rubber stopper (EPDM rubber stopper, WidgetCo, Houston, TX) and brought outside the anaerobic chamber, and 100 μL of 0.1% (wt/vol) *N*,*N*-dimethyl-*p*-phenylenediamine in 20% (vol/vol) H_2_SO_4_ was added to it with a syringe. Then, the rubber stopper of the tube was removed, and 50 μL of 1% (wt/vol) FeCl_3_ in 4% (vol/vol) H_2_SO_4_ was added to the assay mixture. After incubation under air at 25°C for 15 min, the absorbance of the resultant solution was read at 670 nm. Sodium sulfide was used as the standard. From the values of Fe and sulfide (moles per microgram of protein) derived from the above-described assays, and considering that the theoretical subunit mass of the protein is 69.10 kDa, the values for the Fe and acid-labile sulfur contents of ANME2c-FsrII-6D were calculated.

The ammonia produced in an enzymatic reaction mixture was captured and assayed following the above-described protocol for the determination of acid-labile sulfur content, except that for the ammonia assay, the capillary was filled with 1 M HCl, the release of ammonia gas was initiated by the addition of 1 M NaOH to the reaction mixture that was placed in the annular space, and the ammonia concentration in the solution retrieved from the capillary was estimated via a glutamate dehydrogenase-based assay employing a kit (kit AA0100, Sigma-Aldrich).

Protein concentration was estimated via Bradford assay (83) using the dye reagent purchased from Bio-Rad Laboratories (Hercules, CA).

### Bioinformatics methods.

The apparent kinetic constants for the uninhibited reactions catalyzed by ANME2c-FsrII-6D were obtained by fitting the initial velocity data to the Henri-Michaelis-Menten equation: *v* = *V*_max_ [S]/(*K_m_* + [S]) (where *v* is initial velocity, [S] is substrate concentration, *K_m_* is the Michaelis constant, and *V*_max_ is maximum velocity) by using Solver in Microsoft Excel (84). The initial velocity data from inhibition studies were analyzed by fitting to the competitive inhibition model, *v* = *V*_max_ [S]/{*K_m_* (1+[I]/*K_i_*) + [S]} (where [I] is inhibitor concentration and *K_i_* is the inhibition constant), using an R statistical package (85). Theoretical values for the isoelectric points (pIs) of ANME2c-FsrII-6D and *Mj*FsrI were calculated using the ExPASy-ProtParam tool (86).

Multiple-sequence alignment of protein sequences was performed using MUSCLE (87) with default settings and the output was visualized in Jalview 2.11.0 (88). The 3D structures of *Mj*FsrI and ANME2c-FsrII-6D were predicted by using AlphaFold2 in the default setting (35) run on the ColabFold platform (34) (https://colab.research.google.com/github/sokrypton/ColabFold/blob/main/AlphaFold2.ipynb). The predicted structures were visualized using PyMOL (PyMOL molecular graphics system, version 2.3.2; Schrödinger, LLC). Docking of prosthetic groups into the modeled 3D structures of *Mj*FsrI and ANME2c-FsrII-6D were performed as a structural alignment by employing the *align* command line in PyMOL; for the N-terminal half (Fsr-N), the alignment was performed with the Methanothermobacter marburgensis F_420_-reducing [NiFe]-hydrogenase subunit B (FrhB; PDB ID 3ZFS, chain C), and for the C-terminal half (Fsr-C), it was performed with the Archaeoglobus fulgidus dissimilatory sulfite reductase subunit A (DsrA; PDB ID 3MM5, chain A).

## References

[1] Reeburgh WS. 2007. Oceanic methane biogeochemistry. Chem Rev 107:486–513. 10.1021/cr050362v. 17261072

[2] Knittel K, Boetius A. 2009. Anaerobic oxidation of methane: progress with an unknown process. Annu Rev Microbiol 63:311–334. 10.1146/annurev.micro.61.080706.093130. 19575572

[3] Milucka J, Ferdelman TG, Polerecky L, Franzke D, Wegener G, Schmid M, Lieberwirth I, Wagner M, Widdel F, Kuypers MM. 2012. Zero-valent sulphur is a key intermediate in marine methane oxidation. Nature 491:541–546. 10.1038/nature11656. 23135396

[4] Schreiber L, Holler T, Knittel K, Meyerdierks A, Amann R. 2010. Identification of the dominant sulfate-reducing bacterial partner of anaerobic methanotrophs of the ANME-2 clade. Environ Microbiol 12:2327–2340. 10.1111/j.1462-2920.2010.02275.x. 21966923

[5] Chadwick GL, Skennerton CT, Laso-Perez R, Leu AO, Speth DR, Yu H, Morgan-Lang C, Hatzenpichler R, Goudeau D, Malmstrom R, Brazelton WJ, Woyke T, Hallam SJ, Tyson GW, Wegener G, Boetius A, Orphan VJ. 2022. Comparative genomics reveals electron transfer and syntrophic mechanisms differentiating methanotrophic and methanogenic archaea. PLoS Biol 20:e3001508. 10.1371/journal.pbio.3001508. 34986141 PMC9012536

[6] Rinke C, Chuvochina M, Mussig AJ, Chaumeil PA, Davin AA, Waite DW, Whitman WB, Parks DH, Hugenholtz P. 2021. A standardized archaeal taxonomy for the Genome Taxonomy Database. Nat Microbiol 6:946–959. 10.1038/s41564-021-00918-8. 34155373

[7] Wegener G, Krukenberg V, Riedel D, Tegetmeyer HE, Boetius A. 2015. Intercellular wiring enables electron transfer between methanotrophic archaea and bacteria. Nature 526:587–590. 10.1038/nature15733. 26490622

[8] Meyerdierks A, Kube M, Kostadinov I, Teeling H, Glockner FO, Reinhardt R, Amann R. 2010. Metagenome and mRNA expression analyses of anaerobic methanotrophic archaea of the ANME-1 group. Environ Microbiol 12:422–439. 10.1111/j.1462-2920.2009.02083.x. 19878267

[9] Scheller S, Yu H, Chadwick GL, McGlynn SE, Orphan VJ. 2016. Artificial electron acceptors decouple archaeal methane oxidation from sulfate reduction. Science 351:703–707. 10.1126/science.aad7154. 26912857

[10] Skennerton CT, Chourey K, Iyer R, Hettich RL, Tyson GW, Orphan VJ. 2017. Methane-fueled syntrophy through extracellular electron transfer: uncovering the genomic traits conserved within diverse bacterial partners of anaerobic methanotrophic archaea. mBio 8:e01561-17. 10.1128/mBio.01561-17. 28765215 PMC5539420

[11] McGlynn SE, Chadwick GL, Kempes CP, Orphan VJ. 2015. Single cell activity reveals direct electron transfer in methanotrophic consortia. Nature 526:531–535. 10.1038/nature15512. 26375009

[12] McGlynn SE. 2017. Energy metabolism during anaerobic methane oxidation in ANME archaea. Microbes Environ 32:5–13. 10.1264/jsme2.ME16166. 28321009 PMC5371075

[13] He X, Chadwick GL, Kempes CP, Orphan VJ, Meile C. 2021. Controls on interspecies electron transport and size limitation of anaerobically methane-oxidizing microbial consortia. mBio 12:e03620-20. 10.1128/mBio.03620-20. 33975943 PMC8263020

[14] Yu H, Susanti D, McGlynn SE, Skennerton CT, Chourey K, Iyer R, Scheller S, Tavormina PL, Hettich RL, Mukhopadhyay B, Orphan VJ. 2018. Comparative genomics and proteomic analysis of assimilatory sulfate reduction pathways in anaerobic methanotrophic archaea. Front Microbiol 9:2917. 10.3389/fmicb.2018.02917. 30559729 PMC6286981

[15] Yu H, Skennerton CT, Chadwick GL, Leu AO, Aoki M, Tyson GW, Orphan VJ. 2022. Sulfate differentially stimulates but is not respired by diverse anaerobic methanotrophic archaea. ISME J 16:168–177. 10.1038/s41396-021-01047-0. 34285362 PMC8692474

[16] Krukenberg V, Riedel D, Gruber-Vodicka HR, Buttigieg PL, Tegetmeyer HE, Boetius A, Wegener G. 2018. Gene expression and ultrastructure of meso- and thermophilic methanotrophic consortia. Environ Microbiol 20:1651–1666. 10.1111/1462-2920.14077. 29468803 PMC5947290

[17] Milucka J, Widdel F, Shima S. 2013. Immunological detection of enzymes for sulfate reduction in anaerobic methane-oxidizing consortia. Environ Microbiol 15:1561–1571. 10.1111/1462-2920.12003. 23095164

[18] Wang FP, Zhang Y, Chen Y, He Y, Qi J, Hinrichs KU, Zhang XX, Xiao X, Boon N. 2014. Methanotrophic archaea possessing diverging methane-oxidizing and electron-transporting pathways. ISME J 8:1069–1078. 10.1038/ismej.2013.212. 24335827 PMC3996691

[19] Johnson EF, Mukhopadhyay B. 2005. A new type of sulfite reductase, a novel coenzyme F_420_-dependent enzyme, from the methanarchaeon *Methanocaldococcus jannaschii*. J Biol Chem 280:38776–38786. 10.1074/jbc.M503492200. 16048999

[20] Johnson EF, Mukhopadhyay B. 2008. Coenzyme F_420_-dependent sulfite reductase-enabled sulfite detoxification and use of sulfite as a sole sulfur source by *Methanococcus maripaludis*. Appl Environ Microbiol 74:3591–3595. 10.1128/AEM.00098-08. 18378657 PMC2423035

[21] Susanti D, Frazier MC, Mukhopadhyay B. 2019. A genetic system for *Methanocaldococcus jannaschii*: an evolutionary deeply rooted hyperthermophilic methanarchaeon. Front Microbiol 10:1256. 10.3389/fmicb.2019.01256. 31333590 PMC6616113

[22] Susanti D, Mukhopadhyay B. 2012. An intertwined evolutionary history of methanogenic archaea and sulfate reduction. PLoS One 7:e45313. 10.1371/journal.pone.0045313. 23028926 PMC3448663

[23] Johnson EF, Mukhopadhyay B. 2007. A novel coenzyme F_420_-dependent sulfite reductase and a small size sulfite reductase in methanogenic archaea, p 202–216. *In* Dahl C, Friedrich CG (ed), Proceedings of the International Symposium on Microbial Sulfur Metabolism. Springer, New York, NY.

[24] Vigneron A, Alsop EB, Cruaud P, Philibert G, King B, Baksmaty L, Lavallee D, Lomans BP, Eloe-Fadrosh E, Kyrpides NC, Head IM, Tsesmetzis N. 2019. Contrasting pathways for anaerobic methane oxidation in Gulf of Mexico cold seep sediments. mSystems 4:e00091-18. 10.1128/mSystems.00091-18. 30834326 PMC6392090

[25] Moura I, Lino AR, Moura JJ, Xavier AV, Fauque G, Peck HD, Jr, LeGall J. 1986. Low-spin sulfite reductases: a new homologous group of non-heme iron-siroheme proteins in anaerobic bacteria. Biochem Biophys Res Commun 141:1032–1041. 10.1016/S0006-291X(86)80148-6. 3028382

[26] Krueger RJ, Siegel LM. 1982. Spinach siroheme enzymes: isolation and characterization of ferredoxin-sulfite reductase and comparison of properties with ferredoxin-nitrite reductase. Biochemistry 21:2892–2904. 10.1021/bi00541a014. 7104302

[27] Parey K, Warkentin E, Kroneck PM, Ermler U. 2010. Reaction cycle of the dissimilatory sulfite reductase from *Archaeoglobus fulgidus*. Biochemistry 49:8912–8921. 10.1021/bi100781f. 20822098

[28] Wolfe BM, Lui SM, Cowan JA. 1994. Desulfoviridin, a multimeric-dissimilatory sulfite reductase from *Desulfovibrio vulgaris* (Hildenborough). Purification, characterization, kinetics and EPR studies. Eur J Biochem 223:79–89. 10.1111/j.1432-1033.1994.tb18968.x. 8033912

[29] Siegel LM, Davis PS, Kamin H. 1974. Reduced nicotinamide adenine dinucleotide phosphate-sulfite reductase of enterobacteria. 3. The *Escherichia coli* hemoflavoprotein: catalytic parameters and the sequence of electron flow. J Biol Chem 249:1572–1586. 10.1016/S0021-9258(19)42921-9. 4150390

[30] Jackson RH, Cole JA, Cornish-Bowden A. 1982. The steady state kinetics of the NADH-dependent nitrite reductase from *Escherichia coli* K12. The reduction of single-electron acceptors. Biochem J 203:505–510. 10.1042/bj2030505. 6288003 PMC1158256

[31] Kobayashi S, Hira D, Yoshida K, Toyofuku M, Shida Y, Ogasawara W, Yamaguchi T, Araki N, Oshiki M. 2018. Nitric oxide production from nitrite reduction and hydroxylamine oxidation by copper-containing dissimilatory nitrite reductase (NirK) from the aerobic ammonia-oxidizing archaeon, *Nitrososphaera viennensis*. Microbes Environ 33:428–434. 10.1264/jsme2.ME18058. 30318500 PMC6308003

[32] Bruggemann H, Falinski F, Deppenmeier U. 2000. Structure of the F_420_H_2_:quinone oxidoreductase of *Archaeoglobus fulgidus* identification and overproduction of the F_420_H_2_-oxidizing subunit. Eur J Biochem 267:5810–5814. 10.1046/j.1432-1327.2000.01657.x. 10971593

[33] Baumer S, Ide T, Jacobi C, Johann A, Gottschalk G, Deppenmeier U. 2000. The F_420_H_2_ dehydrogenase from *Methanosarcina mazei* is a redox-driven proton pump closely related to NADH dehydrogenases. J Biol Chem 275:17968–17973. 10.1074/jbc.M000650200. 10751389

[34] Mirdita M, Schuetze K, Moriwaki Y, Heo L, Ovchinnikov S, Steinegger M. 2021. ColabFold—making protein folding accessible to all. bioRxiv. 10.1101/2021.08.15.456425. PMC9184281 35637307

[35] Jumper J, Evans R, Pritzel A, Green T, Figurnov M, Ronneberger O, Tunyasuvunakool K, Bates R, Žídek A, Potapenko A, Bridgland A, Meyer C, Kohl SAA, Ballard AJ, Cowie A, Romera-Paredes B, Nikolov S, Jain R, Adler J, Back T, Petersen S, Reiman D, Clancy E, Zielinski M, Steinegger M, Pacholska M, Berghammer T, Bodenstein S, Silver D, Vinyals O, Senior AW, Kavukcuoglu K, Kohli P, Hassabis D. 2021. Highly accurate protein structure prediction with AlphaFold. Nature 596:583–589. 10.1038/s41586-021-03819-2. 34265844 PMC8371605

[36] Oliveira TF, Vonrhein C, Matias PM, Venceslau SS, Pereira IA, Archer M. 2008. The crystal structure of *Desulfovibrio vulgaris* dissimilatory sulfite reductase bound to DsrC provides novel insights into the mechanism of sulfate respiration. J Biol Chem 283:34141–34149. 10.1074/jbc.M805643200. 18829451 PMC2662231

[37] Schiffer A, Parey K, Warkentin E, Diederichs K, Huber H, Stetter KO, Kroneck PM, Ermler U. 2008. Structure of the dissimilatory sulfite reductase from the hyperthermophilic archaeon *Archaeoglobus fulgidus*. J Mol Biol 379:1063–1074. 10.1016/j.jmb.2008.04.027. 18495156

[38] Soo VWC, McAnulty MJ, Tripathi A, Zhu F, Zhang L, Hatzakis E, Smith PB, Agrawal S, Nazem-Bokaee H, Gopalakrishnan S, Salis HM, Ferry JG, Maranas CD, Patterson AD, Wood TK. 2016. Reversing methanogenesis to capture methane for liquid biofuel precursors. Microb Cell Fact 15:11. 10.1186/s12934-015-0397-z. 26767617 PMC4714516

[39] Nauhaus K, Albrecht M, Elvert M, Boetius A, Widdel F. 2007. In vitro cell growth of marine archaeal-bacterial consortia during anaerobic oxidation of methane with sulfate. Environ Microbiol 9:187–196. 10.1111/j.1462-2920.2006.01127.x. 17227423

[40] Holler T, Widdel F, Knittel K, Amann R, Kellermann MY, Hinrichs KU, Teske A, Boetius A, Wegener G. 2011. Thermophilic anaerobic oxidation of methane by marine microbial consortia. ISME J 5:1946–1956. 10.1038/ismej.2011.77. 21697963 PMC3223311

[41] Boone DR, Whitman WB, Rouviere P. 1993. Diversity and taxonomy of methanogens, p 35–80. *In* Ferry JG (ed), Methanogenesis: ecology, physiology, biochemistry and genetics. Chapman and Hall, New York, NY.

[42] Gundry RL, White MY, Murray CI, Kane LA, Fu Q, Stanley BA, Van Eyk JE. 2009. Preparation of proteins and peptides for mass spectrometry analysis in a bottom-up proteomics workflow. Curr Protoc Mol Biol Chapter 10:Unit10 25. 10.1002/0471142727.mb1025s88. PMC2905857 19816929

[43] Timmers PH, Welte CU, Koehorst JJ, Plugge CM, Jetten MS, Stams AJ. 2017. Reverse methanogenesis and respiration in methanotrophic archaea. Archaea 2017:1654237. 10.1155/2017/1654237. 28154498 PMC5244752

[44] Matthews JC, Timkovich R, Liu MY, Le Gall J. 1995. Siroamide: a prosthetic group isolated from sulfite reductases in the genus *Desulfovibrio*. Biochemistry 34:5248–5251. 10.1021/bi00015a039. 7711045

[45] Lübbe YJ, Youn H-S, Timkovich R, Dahl C. 2006. Siro(haem)amide in *Allochromatium vinosum* and relevance of DsrL and DsrN, a homolog of cobyrinic acid a,c-diamide synthase, for sulphur oxidation. FEMS Microbiol Lett 261:194–202. 10.1111/j.1574-6968.2006.00343.x. 16907720

[46] Moura JJ, Moura I, Santos H, Xavier AV, Scandellari M, LeGall J. 1982. Isolation of P590 from *Methanosarcina barkeri*: evidence for the presence of sulfite reductase activity. Biochem Biophys Res Commun 108:1002–1009. 10.1016/0006-291x(82)92099-x. 7181874

[47] Brockman KL, Shirodkar S, Croft TJ, Banerjee R, Saffarini DA. 2020. Regulation and maturation of the *Shewanella oneidensis* sulfite reductase SirA. Sci Rep 10:953. 10.1038/s41598-020-57587-6. 31969587 PMC6976685

[48] Abken HJ, Deppenmeier U. 1997. Purification and properties of an F_420_H_2_ dehydrogenase from *Methanosarcina mazei* Go1. FEMS Lett 154:231–237. 10.1016/S0378-1097(97)00330-3.

[49] Susanti D, Loganathan U, Mukhopadhyay B. 2016. A novel F_420_-dependent thioredoxin reductase gated by low potential FAD: a tool for redox regulation in an anaerobe. J Biol Chem 291:23084–23100. 10.1074/jbc.M116.750208. 27590343 PMC5087728

[50] Kern M, Volz J, Simon J. 2011. The oxidative and nitrosative stress defence network of *Wolinella succinogenes*: cytochrome c nitrite reductase mediates the stress response to nitrite, nitric oxide, hydroxylamine and hydrogen peroxide. Environ Microbiol 13:2478–2494. 10.1111/j.1462-2920.2011.02520.x. 21672122

[51] Sivan O, Antler G, Turchyn AV, Marlow JJ, Orphan VJ. 2014. Iron oxides stimulate sulfate-driven anaerobic methane oxidation in seeps. Proc Natl Acad Sci USA 111:E4139–E4147. 10.1073/pnas.1412269111. 25246590 PMC4209987

[52] Holmkvist L, Ferdelman TG, Jørgensen BB. 2011. A cryptic sulfur cycle driven by iron in the methane zone of marine sediment (Aarhus Bay, Denmark). Geochim Cosmochim Acta 75:3581–3599. 10.1016/j.gca.2011.03.033.

[53] Becker DF, Ragsdale SW. 1998. Activation of methyl-SCoM reductase to high specific activity after treatment of whole cells with sodium sulfide. Biochemistry 37:2639–2647. 10.1021/bi972145x. 9485414

[54] Mahlert F, Bauer C, Jaun B, Thauer RK, Duin EC. 2002. The nickel enzyme methyl-coenzyme M reductase from methanogenic archaea: in vitro induction of the nickel-based MCR-ox EPR signals from MCR-red2. J Biol Inorg Chem 7:500–513. 10.1007/s00775-001-0325-z. 11941508

[55] Duin EC, Wagner T, Shima S, Prakash D, Cronin B, Yanez-Ruiz DR, Duval S, Rumbeli R, Stemmler RT, Thauer RK, Kindermann M. 2016. Mode of action uncovered for the specific reduction of methane emissions from ruminants by the small molecule 3-nitrooxypropanol. Proc Natl Acad Sci USA 113:6172–6177. 10.1073/pnas.1600298113. 27140643 PMC4896709

[56] Duin EC, Signor L, Piskorski R, Mahlert F, Clay MD, Goenrich M, Thauer RK, Jaun B, Johnson MK. 2004. Spectroscopic investigation of the nickel-containing porphinoid cofactor F_430_. Comparison of the free cofactor in the +1, +2 and +3 oxidation states with the cofactor bound to methyl-coenzyme M reductase in the silent, red and ox forms. J Biol Inorg Chem 9:563–576. 10.1007/s00775-004-0549-9. 15160314

[57] Bowles M, Joye S. 2011. High rates of denitrification and nitrate removal in cold seep sediments. ISME J 5:565–567. 10.1038/ismej.2010.134. 20944683 PMC3105719

[58] Russ L, Kartal B, Op den Camp HJM, Sollai M, Le Bruchec J, Caprais J-C, Godfroy A, Sinninghe Damsté JS, Jetten MSM. 2013. Presence and diversity of anammox bacteria in cold hydrocarbon-rich seeps and hydrothermal vent sediments of the Guaymas Basin. Front Microbiol 4:219. 10.3389/fmicb.2013.00219. 23935595 PMC3731535

[59] Haroon MF, Hu S, Shi Y, Imelfort M, Keller J, Hugenholtz P, Yuan Z, Tyson GW. 2013. Anaerobic oxidation of methane coupled to nitrate reduction in a novel archaeal lineage. Nature 500:567–570. 10.1038/nature12375. 23892779

[60] Arshad A, Speth DR, de Graaf RM, Op den Camp HJM, Jetten MSM, Welte CU. 2015. A metagenomics-based metabolic model of nitrate-dependent anaerobic oxidation of methane by Methanoperedens-like archaea. Front Microbiol 6:1423. 10.3389/fmicb.2015.01423. 26733968 PMC4683180

[61] Jones CG, Martynowycz MW, Hattne J, Fulton TJ, Stoltz BM, Rodriguez JA, Nelson HM, Gonen T. 2018. The CryoEM method MicroED as a powerful tool for small molecule structure determination. ACS Cent Sci 4:1587–1592. 10.1021/acscentsci.8b00760. 30555912 PMC6276044

[62] Guss AM, Rother M, Zhang JK, Kulkarni G, Metcalf WW. 2008. New methods for tightly regulated gene expression and highly efficient chromosomal integration of cloned genes for *Methanosarcina* species. Archaea 2:193–203. 10.1155/2008/534081. 19054746 PMC2685592

[63] Metcalf WW, Zhang JK, Shi X, Wolfe RS. 1996. Molecular, genetic, and biochemical characterization of the serC gene of *Methanosarcina barkeri* Fusaro. J Bacteriol 178:5797–5802. 10.1128/jb.178.19.5797-5802.1996. 8824630 PMC178424

[64] Metcalf WW, Zhang JK, Apolinario E, Sowers KR, Wolfe RS. 1997. A genetic system for archaea of the genus *Methanosarcina*: liposome-mediated transformation and construction of shuttle vectors. Proc Natl Acad Sci USA 94:2626–2631. 10.1073/pnas.94.6.2626. 9122246 PMC20139

[65] Haase P, Deppenmeier U, Blaut M, Gottschalk G. 1992. Purification and characterization of F_420_H_2_-dehydrogenase from *Methanolobus tindarius*. Eur J Biochem 203:527–531. 10.1111/j.1432-1033.1992.tb16579.x. 1735436

[66] Purwantini E, Daniels L. 1996. Purification of a novel coenzyme F_420_-dependent glucose-6-phosphate dehydrogenase from *Mycobacterium smegmatis*. J Bacteriol 178:2861–2866. 10.1128/jb.178.10.2861-2866.1996. 8631674 PMC178021

[67] Laemmli UK. 1970. Cleavage of structural proteins during the assembly of the head of bacteriophage T4. Nature 227:680–685. 10.1038/227680a0. 5432063

[68] Shevchenko A, Tomas H, Havli J, Olsen JV, Mann M. 2006. In-gel digestion for mass spectrometric characterization of proteins and proteomes. Nat Protoc 1:2856–2860. 10.1038/nprot.2006.468. 17406544

[69] Eliuk S, Makarov A. 2015. Evolution of Orbitrap mass spectrometry instrumentation. Annu Rev Anal Chem 8:61–80. 10.1146/annurev-anchem-071114-040325. 26161972

[70] Wang Y, Ahn T-H, Li Z, Pan C. 2013. Sipros/ProRata: a versatile informatics system for quantitative community proteomics. Bioinformatics 29:2064–2065. 10.1093/bioinformatics/btt329. 23793753

[71] Galagan JE, Nusbaum C, Roy A, Endrizzi MG, Macdonald P, FitzHugh W, Calvo S, Engels R, Smirnov S, Atnoor D, Brown A, Allen N, Naylor J, Stange-Thomann N, DeArellano K, Johnson R, Linton L, McEwan P, McKernan K, Talamas J, Tirrell A, Ye W, Zimmer A, Barber RD, Cann I, Graham DE, Grahame DA, Guss AM, Hedderich R, Ingram-Smith C, Kuettner HC, Krzycki JA, Leigh JA, Li W, Liu J, Mukhopadhyay B, Reeve JN, Smith K, Springer TA, Umayam LA, White O, White RH, Conway de Macario E, Ferry JG, Jarrell KF, Jing H, Macario AJL, Paulsen I, Pritchett M, Sowers KR, et al. 2002. The genome of *M. acetivorans* reveals extensive metabolic and physiological diversity. Genome Res 12:532–542. 10.1101/gr.223902. 11932238 PMC187521

[72] Elias JE, Gygi SP. 2010. Target-decoy search strategy for mass spectrometry-based proteomics. Methods Mol Biol 604:55–71. 10.1007/978-1-60761-444-9_5. 20013364 PMC2922680

[73] Mukhopadhyay B, Purwantini E. 2000. Pyruvate carboxylase from *Mycobacterium smegmatis*: stabilization, rapid purification, molecular and biochemical characterization and regulation of the cellular level. Biochim Biophys Acta 1475:191–206. 10.1016/s0304-4165(00)00064-7. 10913817

[74] Gliszczyńska A, Koziołowa A. 1998. Chromatographic determination of flavin derivatives in baker’s yeast. J Chromatogr A 822:59–66. 10.1016/S0021-9673(98)00557-3. 9810711

[75] Purwantini E, Loganathan U, Mukhopadhyay B. 2018. Coenzyme F_420_-dependent glucose-6-phosphate dehydrogenase-coupled polyglutamylation of coenzyme F_420_ in mycobacteria. J Bacteriol 200:e00375-18. 10.1128/JB.00375-18. 30249701 PMC6222201

[76] Mukhopadhyay B, Daniels L. 1989. Aerobic purification of N^5^,N^10^-methylenetetrahydromethanopterin dehydrogenase, separated from N^5^,N^10^-methylenetetrahydromethanopterin cyclohydrolase, from *Methanobacterium thermoautotrophicum* strain Marburg. Can J Microbiol 35:499–507. 10.1139/m89-077. 2743220

[77] Jacobson FS, Daniels L, Fox JA, Walsh CT, Orme-Johnson WH. 1982. Purification and properties of an 8-hydroxy-5-deazaflavin-reducing hydrogenase from *Methanobacterium thermoautotrophicum*. J Biol Chem 257:3385–3388. 10.1016/S0021-9258(18)34788-4. 7061485

[78] Purwantini E, Mukhopadhyay B, Spencer RW, Daniels L. 1992. Effect of temperature on the spectral properties of coenzyme F_420_ and related compounds. Anal Biochem 205:342–350. 10.1016/0003-2697(92)90446-e. 1443583

[79] Pachmayr F. 1960. Vorkommen und Bestimmung von Schwefelverbindungen in Mineralwasser. Ph.D. thesis. University of Munich, Munich, Germany.

[80] Trueper HG, Schlegel HG. 1964. Sulphur metabolism in Thiorhodaceae. I. Quantitative measurements on growing cells of *Chromatium okenii*. Antonie Van Leeuwenhoek 30:225–238. 10.1007/BF02046728. 14218435

[81] Bouda J. 1968. Determination of iron with bathophenanthroline without deproteinisation. Clin Chim Acta 21:159–160. 10.1016/0009-8981(68)90026-0. 5658949

[82] Tchong SI, Xu H, White RH. 2005. L-cysteine desulfidase: an [4Fe-4S] enzyme isolated from *Methanocaldococcus jannaschii* that catalyzes the breakdown of L-cysteine into pyruvate, ammonia, and sulfide. Biochemistry 44:1659–1670. 10.1021/bi0484769. 15683250

[83] Bradford MM. 1976. A rapid and sensitive method for the quantitation of microgram quantities of protein utilizing the principle of protein-dye binding. Anal Biochem 72:248–254. 10.1006/abio.1976.9999. 942051

[84] Kemmer G, Keller S. 2010. Nonlinear least-squares data fitting in Excel spreadsheets. Nat Protoc 5:267–281. 10.1038/nprot.2009.182. 20134427

[85] R-Core-Team. 2012. R: a language and environment for statistical computing. Foundation for Statistical Computing, Vienna, Austria.

[86] Gasteiger EHC, Gattiker A, Duvaud S, Wilkins MR, Appel RD, Bairoch A. 2005. Protein identification and analysis tools on the ExPASy server, p 571–607. *In* Walker JM (ed), The proteomics protocols handbook. Humana Press, Totowa, NJ.

[87] Edgar RC. 2004. MUSCLE: multiple sequence alignment with high accuracy and high throughput. Nucleic Acids Res 32:1792–1797. 10.1093/nar/gkh340. 15034147 PMC390337

[88] Waterhouse AM, Procter JB, Martin DM, Clamp M, Barton GJ. 2009. Jalview Version 2—a multiple sequence alignment editor and analysis workbench. Bioinformatics 25:1189–1191. 10.1093/bioinformatics/btp033. 19151095 PMC2672624

[89] Mills DJ, Vitt S, Strauss M, Shima S, Vonck J. 2013. De novo modeling of the F_420_-reducing [NiFe]-hydrogenase from a methanogenic archaeon by cryo-electron microscopy. Elife 2:e00218. 10.7554/eLife.00218. 23483797 PMC3591093

[90] Dym O, Eisenberg D. 2001. Sequence-structure analysis of FAD-containing proteins. Protein Sci 10:1712–1728. 10.1110/ps.12801. 11514662 PMC2253189

[91] van den Heuvel RH, Curti B, Vanoni MA, Mattevi A. 2004. Glutamate synthase: a fascinating pathway from L-glutamine to L-glutamate. Cell Mol Life Sci 61:669–681. 10.1007/s00018-003-3316-0. 15052410 PMC11138638

[92] Shuber AP, Orr EC, Recny MA, Schendel PF, May HD, Schauer NL, Ferry JG. 1986. Cloning, expression, and nucleotide sequence of the formate dehydrogenase genes from *Methanobacterium formicicum*. J Biol Chem 261:12942–12947. 3531194

