# Supplementary material for: A Reduced F420-Dependent Nitrite Reductase in an Anaerobic Methanotrophic Archaeon
Source: J Bacteriol. 2022 Jun 13;204(7):e00078-22. doi: 10.1128/jb.00078-22 (PMC9295563; doi:10.1128/jb.00078-22)
Supplement: Supplemental file 1 — Supplemental material. Download jb.00078-22-s0001.pdf, PDF file, 2.0 MB [file jb.00078-22-s0001.pdf]

Supporting Information

For

A reduced F_{420}-dependent nitrite reductase (FNiR) in an anaerobic methanotrophic archaeon

Christian Heryakusuma †[1,2], Dwi Susanti†[2], Hang Yu[3], Zhou Li[4], Endang Purwantini[2], Robert L. Hettich[4], Victoria J. Orphan[3]*, Biswarup Mukhopadhyay[2,5]*

[1]Genetics, Bioinformatics, and Computational Biology Ph.D. Program, Virginia Tech, Blacksburg, VA 24061, USA

[2]Department of Biochemistry, Virginia Tech, Blacksburg, VA 24061, USA

[3]Division of Geological and Planetary Sciences, California Institute of Technology, Pasadena, CA 91125, USA

[4]Biosciences Division, Oak Ridge National Laboratory, Oak Ridge, TN 37831, USA

[5]Virginia Tech Carilion School of Medicine, Virginia Tech, Blacksburg, VA 24061, USA

†These authors contributed equally to this work.

Author contributions: all authors designed research; C.H., D.S., H.Y., Z.L., and E.P. performed research; all authors analyzed data; C.H., D.S., H.Y., V.J.O., and B.M. wrote the paper.

***Correspondence:**

Biswarup Mukhopadhyay, Department of Biochemistry, Virginia Tech, Engel Hall, 340 West Campus Drive, Blacksburg, VA 24061; Phone: 540 231 1219; E-mail: biswarup@vt.edu

Victoria J. Orphan, Division of Geological and Planetary Sciences, Caltech, Mail code 100-23, 1200 E. California Blvd., Pasadena, CA 91125; E-mail: vorphan@gps.caltech.edu

Contents	Page
Materials and Methods	S1
FIGURE S1	S2
FIGURE S2	S3
Table S1	S4

MATERIALS AND METHODS

Construction of a Methanosarcina acetivorans strain expressing ANME FsrII

The coding sequence for FsrII was previously PCR amplified from the metagenomic DNA, that was extracted from an ANME-2 dominated methane seep sediment, and cloned into the plasmid pCR4-TOPO (1). For the current study, from one such clone, called Fsr-5207-6D, the coding sequence was further amplified using the following primers (restriction sites underlined and names presented within the parentheses): forward primer, 5'-AAGGAGGAAATTCATATGATGGCAAACGAAGAATATAA-3' (*Nde*I); reverse primer, 5'-GAAGTTATCAAGAAGCTTTCACACCTGATCCAGAACCTC-3' (*Hind*III). The amplicon was then assembled with pJK027A (2), that was linearized via digestion with *Nde*I and *Hind*III, employing the In-Fusion® HD Cloning assembly kit (Clontech, Mountainview, CA). In the resulting plasmid, pDS601, the FsrII coding sequence was placed under the control of the *Methanosarcina barkeri mcrB* promoter that was fused with the tetracycline operator (P_{mcrB}(tetO1)) (2); pDS601 was propagated in *E. coli* WWM4489 under chloramphenicol selection (3). Then pDS601 carrying λattB sequence was combined with pAMG40 (2), an *E. coli-Methanosarcina* shuttle vector that contained a λattP site, using BP clonase (Invitrogen, Carlsbad, CA). The fusion plasmid, called pDS701, was transformed into *E. coli* WWM4489, and the transformants were selected on LB agar plates containing 20 μg/mL kanamycin and 34 μg/mL chloramphenicol. Finally, pDS701 was introduced into *M. acetivorans* using a liposome-mediated transformation method and the transformant was selected on HS-TMA solid media containing puromycin (4).

Size exclusion chromatography

The size exclusion chromatography was performed as described previously (5, 6), employing a 7.8-mm × 30-cm TSK-GEL G3000SWXL column (TosoHaas, Montgomeryville, PA), a 6-mm × 4-cm SWxl guard column (TosoHaas), a Shimadzu Prominence HPLC system consisting of LC-20AD dual pumps, SIL-20A autosampler, SPD-M20A diode array detector, and CBM 20A controller system (Shimadzu Scientific Instruments, Columbia, MD), and the calibration standards that are listed in the legend of Fig. 1B. A 20 μL anaerobic solution of 20 μg ANME2c-FsrII-6D protein in 100 mM potassium phosphate buffer, pH 7, was analyzed. The elution was monitored at 280 nm. The UV-visible spectrum collected by using the diode array detector showed that the ANME2c-FsrII-6D was stable during the short duration of chromatographic analysis that employed an aerobic aqueous solution containing 100 mM potassium phosphate buffer pH 7, and 100 mM NaCl as the isocratic mobile phase.

Figure S1

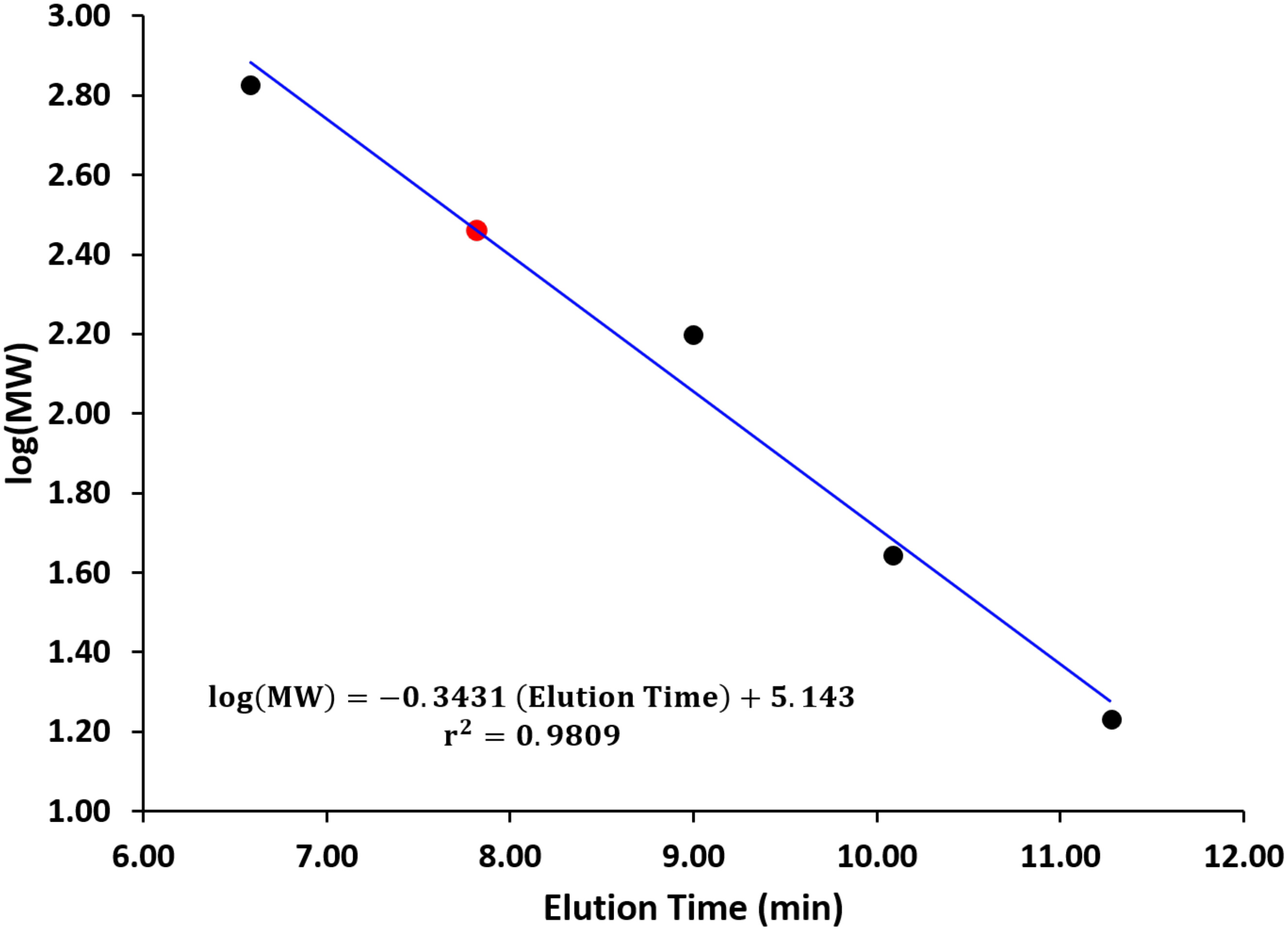

Figure S1. A calibration plot for size-exclusion chromatography. The black dots represent the calibration standards of a, b, c, and d as shown in the inset of Fig. 1B. The apparent globular molecular mass of native ANME2c-FsrII-6D was estimated to be 289.44 kDa. The fitted location of ANME2c-FsrII-6D is shown with a red dot.

Figure S2

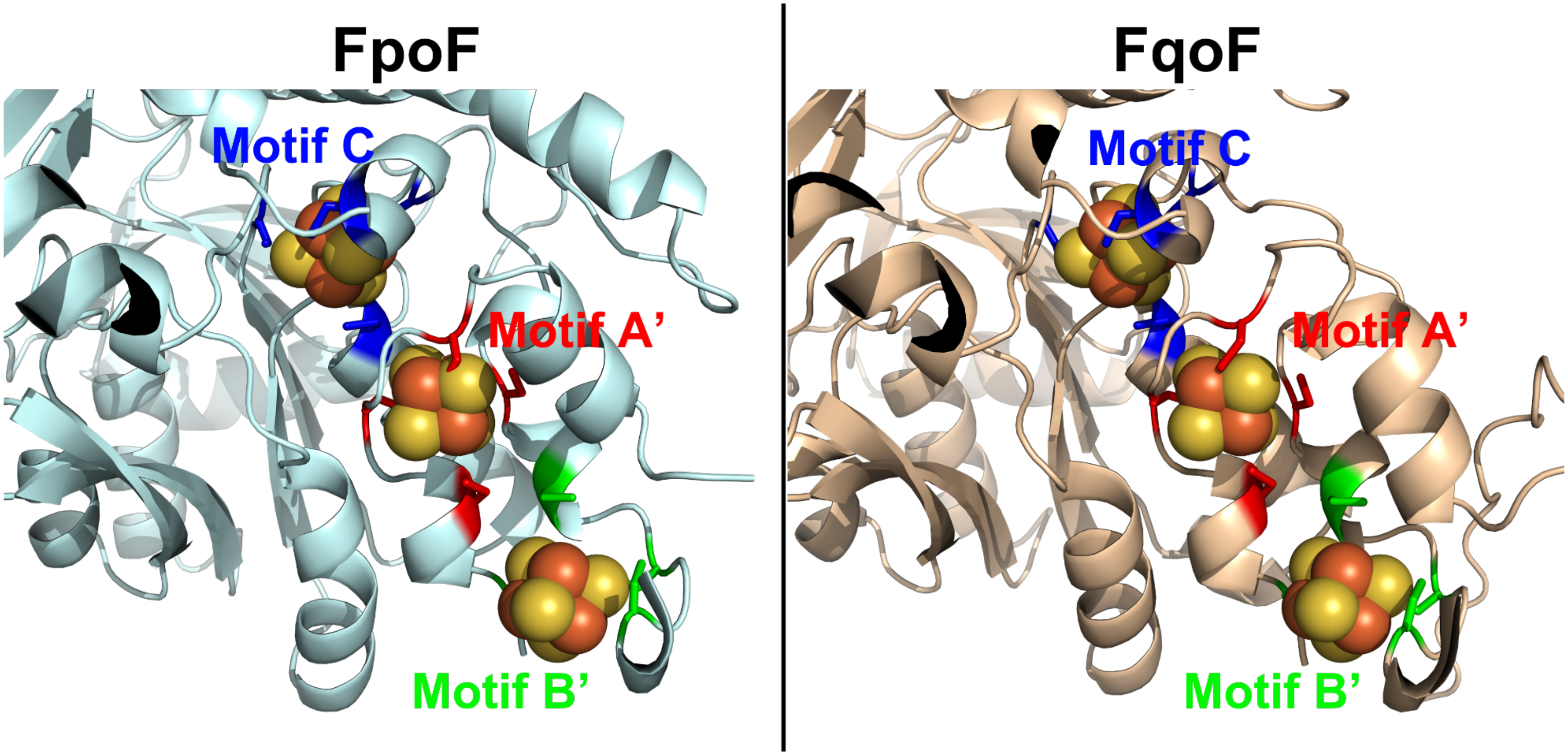

Figure S3. Iron-sulfur cluster binding residues of FpoF and FqoF based on AlphaFold2 model prediction. Modeled structures of FpoF of *M. mazei* and FqoF of *A. fulgidus* with docked [Fe_4-S_4] cluster from *M. marburgensis* F_{420}-reducing [NiFe]-hydrogenase (Frh, PDB ID: 3ZFS) (7). Cysteine residues of the motifs A', B' and C as shown in Fig. 4, are displayed in red, green, and blue colors, respectively.

Table S1. Catalytic Properties of siroheme-dependent nitrite/sulfite reductases.

Organisms (Archaeon, A; Bacterium, B; Plant, P)	Protein Name (NCBI ORF Number)	Reaction	Electron Donor		Electron Acceptor		Refs.
			Electron Donor	K_m (μM)	Electron Acceptor	K_m (μM)	
ANME-2c (A)	ANME2c-FsrII-6D (MH823235)	$3F_{420}H_2 + NO_2^- + 2H^+ \rightarrow 3F_{420} + NH_4^+ + 2H_2O$	$F_{420}H_2$	13.9 ± 2.4 (with DTT)	NO_2^-	4.2 ± 0.5 (with DTT)	This study
		$F_{420}H_2 + NH_2OH + H^+ \rightarrow F_{420} + NH_4^+ + H_2O$	$F_{420}H_2$	ND	NH_2OH	10.5 ± 1.3 (with DTT)	This study
		$F_{420}H_2$ + ($2MV^{2+}$ + Metro) $\rightarrow$ F_{420} + ($2MV^{\bullet+}$ + Metro) + $2H^+$	$F_{420}H_2$	4.7 ± 0.8 (with DTT)	MV^{2+}	ND	This study
		$3F_{420}H_2 + HSO_3^- \nrightarrow 3F_{420} + HS^- + 3H_2O$	$F_{420}H_2$	NR	HSO_3^-	NR	This study
		$6MV^{\bullet+} + HSO_3^- + 6H^+ \rightarrow 6MV^{2+} + HS^- + 3H_2O$	$MV^{\bullet+}$	136.5 ± 26.3 (with DTT)	HSO_3^-	129.2 ± 19.8 (with DTT)	This study
Methanocaldococcus jannaschii (A)	DFTR (Mj_1536)	$F_{420}H_2$ + *Mj*$Trx1_{ox}$ $\rightarrow$ F_{420} + *Mj*$Trx1_{red}$	$F_{420}H_2$	28.6 ± 2.5	*Mj*Trx1	6.3 ± 2	(6)
	Fsr (Mj_0870)	$3F_{420}H_2 + HSO_3^- \rightarrow 3F_{420} + HS^- + 3H_2O$	$F_{420}H_2$	21.2 ± 3.8	HSO_3^-	12.2 ± 1	(8)
Archaeoglobus fulgidus (A)	Dsr (Af_0423-4)	$6MV^{\bullet+} + HSO_3^- + 6H^+ \rightarrow 6MV^{2+} + HS^- + 3H_2O$	$MV^{\bullet+}$	ND	HSO_3^-	ND	(9, 10)
	FqoF (Af_1833)	$F_{420}H_2$ + ($2MV^{2+}$ + Metro) $\rightarrow$ F_{420} + ($2MV^{\bullet+}$ + Metro) + $2H^+$	$F_{420}H_2$	ND	MV^{2+}	ND	(11)
		$F_{420}H_2$ + DMN $\rightarrow$ F_{420} + $DMNH_2$	$F_{420}H_2$	50	DMN	190	(12)
Desulfovibrio vulgaris (B)	Dsr (Dvu_0402-3)	$6MV^{\bullet+} + HSO_3^- + 6H^+ \rightarrow 6MV^{2+} + HS^- + 3H_2O$	$MV^{\bullet+}$	ND	HSO_3^-	60	(13)
		$6MV^{\bullet+} + NO_2^- + 8H^+ \rightarrow 6MV^{2+} + NH_4^+ + 2H_2O$	$MV^{\bullet+}$	ND	NO_2^-	28	(13)
		$2MV^{\bullet+} + NH_2OH + 3H^+ \rightarrow 2MV^{2+} + NH_4^+ + H_2O$	$MV^{\bullet+}$	ND	NH_2OH	4800	(13)

Organisms (Archaeon, A; Bacterium, B; Plant, P)	Protein Name (NCBI ORF Number)	Reaction	Electron Donor		Electron Acceptor		Refs.
			Electron Donor	K_m (µM)	Electron Acceptor	K_m (µM)	
Methanosarcina mazei (A)	FpoF (Mm_0627)	$F_{420}H_2 + (2MV^{2+} + Metro) \rightarrow F_{420} + (2MV^{\bullet+} + Metro) + 2H^+$	$F_{420}H_2$	7	MV^{2+}	ND	(14)
Escherichia coli (B)	SiR (ECK2758-9)	$3NADPH + HSO_3^- + 3H^+ \rightarrow 3NADP^+ + HS^- + 3H_2O$	NADPH	4.5	HSO_3^-	4.3	(15)
		$3NADPH + NO_2^- + 5H^+ \rightarrow 3NADP^+ + NH_4^+ + 2H_2O$	NADPH	26	NO_2^-	800	(15)
		$NADPH + NH_2OH + 2H^+ \rightarrow NADP^+ + NH_4^+ + H_2O$	NADPH	53	NH_2OH	10300	(15)
	NrfA (ECK4063)	$6MV^{\bullet+} + NO_2^- + 8H^+ \rightarrow 6MV^{2+} + NH_4^+ + 2H_2O$	$MV^{\bullet+}$	ND	NO_2^-	22	(16)
	NirB (ECK3353)	$3NADH + NO_2^- + 5H^+ \rightarrow 3NAD^+ + NH_4^+ + 2H_2O$	NADH	ND	NO_2^-	11	(17)
		$NADH + NH_2OH + 2H^+ \rightarrow NAD^+ + NH_4^+ + H_2O$	NADH	ND	NH_2OH	2910	(17)
		$NADH + 2cyt\ c_{ox} \rightarrow NAD^+ + 2cyt\ c_{red} + H^+$	NADH	48.8	Cyt c	ND	(17)
Spinacia oleracea (P)	Fd-NiR (LOC110805491)	$6Fd_{red} + NO_2^- + 8H^+ \rightarrow 6Fd_{ox} + NH_4^+ + 2H_2O$	Fd	33	NO_2^-	17	(18)
		$6MV^{\bullet+} + NO_2^- + 8H^+ \rightarrow 6MV^{2+} + NH_4^+ + 2H_2O$	$MV^{\bullet+}$	ND	NO_2^-	1.3	(18)
Desulfovibrio desulfuricans (B)	NiR (NA)	$6MV^{\bullet+} + NO_2^- + 8H^+ \rightarrow 6MV^{2+} + NH_4^+ + 2H_2O$	$MV^{\bullet+}$	ND	NO_2^-	1140	(19)

Organisms (Archaeon, A; Bacterium, B; Plant, P)	Protein Name (NCBI ORF Number)	Reaction	Electron Donor		Electron Acceptor		Refs.
			Electron Donor	K_m (μM)	Electron Acceptor	K_m (μM)	
Arabidopsis thaliana (P)	NIA1 (AT1G77760)	$3MV^{\bullet+} + NO_3^- + 4H^+ \rightarrow 3MV^{2+} + NO + 2H_2O$	$MV^{\bullet+}$	ND	NO_3^-	2120 ± 160	(20)
		$3NADH + 2NO_3^- + 5H^+ \rightarrow 3NAD^+ + 2NO + 4H_2O$	NADH	ND	NO_3^-	17	(20)
		$BV^{\bullet+} + NO_2^- + 2H^+ \rightarrow BV^{2+} + NO + H_2O$	$BV^{\bullet+}$	ND	NO_2^-	35.5 ± 2.7	(20)
	NIA2 (AT1G37130)	$3MV^{\bullet+} + NO_3^- + 4H^+ \rightarrow 3MV^{2+} + NO + 2H_2O$	$MV^{\bullet+}$	ND	NO_3^-	443 ± 26	(20)
		$3NADH + 2NO_3^- + 5H^+ \rightarrow 3NAD^+ + 2NO + 4H_2O$	NADH	ND	NO_3^-	35	(20)
		$BV^{\bullet+} + NO_2^- + 2H^+ \rightarrow BV^{2+} + NO + H_2O$	$BV^{\bullet+}$	ND	NO_2^-	13.7 ± 3.3	(20)
Candidatus Methylomirabilis lanthanidiphila (B)	NiR (NA)	$3CH_4 + 8NO_2^- + 8H^+ \rightarrow 3CO_2 + 4N_2 + 10H_2O$	CH_4	2.6	NO_2^-	7	(21)
Haloferax mediterranei (A)	NiR (HFX_2005, HFX_1918)	$6MV^{\bullet+} + NO_2^- + 8H^+ \rightarrow 6MV^{2+} + NH_4^+ + 2H_2O$	$MV^{\bullet+}$	1900 ± 200	NO_2^-	8600 ± 200	(22)
Nitrososphaera viennensis (A)	NiR (NVIE_019250)	$6BV^{\bullet+} + NO_2^- + 8H^+ \rightarrow 6BV^{2+} + NH_4^+ + 2H_2O$	$BV^{\bullet+}$	ND	NO_2^-	287	(23)
		$2BV^{\bullet+} + NH_2OH + 3H^+ \rightarrow 2BV^{2+} + NH_4^+ + H_2O$	$BV^{\bullet+}$	ND	NH_2OH	97	(23)

Abbreviation: K_m, Michaelis-Menten constant in μM; ND, not determined; NR, no reaction; NA, not available; DMN, 2,3-dimethyl-1,4-naphthoquinone; Fd, ferredoxin; $F_{420}H_2$, reduced cofactor F_{420}; F_{420}, oxidized cofactor F_{420}; Metro, metronidazole; $MV^{\bullet+}$, reduced methyl viologen; MV^{2+}, oxidized methyl viologen; $BV^{\bullet+}$, reduced benzyl viologen; BV^{2+}, oxidized benzyl viologen; Cyt c, cytochrome c; DFTR, deazaflavin-dependent flavin-containing thioredoxin reductase; Fsr, F_{420}-dependent sulfite reductase; Trx, thioredoxin; Dsr, dissimilatory sulfite reductase; FqoF, F_{420}:quinone oxidoreductase subunit F; FpoF, F_{420}:phenazine oxidoreductase subunit F; SiR, sulfite reductase; NrfA, cytochrome c-dependent nitrite reductase subunit A; NirB, NADH-dependent nitrite reductase subunit B; Fd-NiR, Ferredoxin-dependent nitrite reductase.

REFERENCES

1. Yu H, Susanti D, McGlynn SE, Skennerton CT, Chourey K, Iyer R, Scheller S, Tavormina PL, Hettich RL, Mukhopadhyay B, Orphan VJ. 2018. Comparative Genomics and Proteomic Analysis of Assimilatory Sulfate Reduction Pathways in Anaerobic Methanotrophic Archaea. Front Microbiol 9:2917.
2. Guss AM, Rother M, Zhang JK, Kulkarni G, Metcalf WW. 2008. New methods for tightly regulated gene expression and highly efficient chromosomal integration of cloned genes for *Methanosarcina* species. Archaea 2:193-203.
3. Eliot AC, Griffin BM, Thomas PM, Johannes TW, Kelleher NL, Zhao H, Metcalf WW. 2008. Cloning, expression, and biochemical characterization of *Streptomyces rubellomurinus* genes required for biosynthesis of antimalarial compound FR900098. Chem Biol 15:765-70.
4. Metcalf WW, Zhang JK, Apolinario E, Sowers KR, Wolfe RS. 1997. A genetic system for Archaea of the genus Methanosarcina: liposome-mediated transformation and construction of shuttle vectors. Proc Natl Acad Sci U S A 94:2626-31.
5. Mukhopadhyay B, Purwantini E. 2000. Pyruvate carboxylase from *Mycobacterium smegmatis*: stabilization, rapid purification, molecular and biochemical characterization and regulation of the cellular level. Biochim Biophys Acta 1475:191-206.
6. Susanti D, Loganathan U, Mukhopadhyay B. 2016. A Novel F_{420}-dependent Thioredoxin Reductase Gated by Low Potential FAD: A Tool For Redox Regulation in an Anaerobe. J Biol Chem 291:23084-23100.
7. Mills DJ, Vitt S, Strauss M, Shima S, Vonck J. 2013. De novo modeling of the F_{420}-reducing [NiFe]-hydrogenase from a methanogenic archaeon by cryo-electron microscopy. Elife 2:e00218.
8. Johnson EF, Mukhopadhyay B. 2005. A new type of sulfite reductase, a novel coenzyme F_{420}-dependent enzyme, from the methanarchaeon *Methanocaldococcus jannaschii*. J Biol Chem 280:38776-86.
9. Dahl C, Speich N, Trüper HG. 1994. [23] Enzymology and molecular biology of sulfate reduction in extremely thermophilic archaeon *Archaeoglobus fulgidus*. Methods Enzymol 243:331-349.
10. Dahl C, Trüper HG. 2001. [33] Sulfite reductase and APS reductase from *Archaeoglobus fulgidus*. 331:427-441.
11. Bruggemann H, Falinski F, Deppenmeier U. 2000. Structure of the $F_{420}H_2$:quinone oxidoreductase of *Archaeoglobus fulgidus* identification and overproduction of the $F_{420}H_2$-oxidizing subunit. Eur J Biochem 267:5810-4.
12. Kunow J, Linder D, Stetter KO, Thauer RK. 1994. $F_{420}H_2$:quinone oxidoreductase from *Archaeoglobus fulgidus*. Characterization of a membrane-bound multisubunit complex containing FAD and iron-sulfur clusters. European Journal of Biochemistry 223:503-511.
13. Wolfe BM, Lui SM, Cowan JA. 1994. Desulfoviridin, a multimeric-dissimilatory sulfite reductase from *Desulfovibrio vulgaris* (Hildenborough). Purification, characterization, kinetics and EPR studies. European Journal of Biochemistry 223:79-89.
14. Abken H-J, Deppenmeier U. 1997. Purification and properties of an $F_{420}H_2$ dehydrogenase from *Methanosarcina mazei* Gö1. FEMS Microbiology Letters 154:231-237.
15. Siegel LM, Davis PS, Kamin H. 1974. Reduced nicotinamide adenine dinucleotide phosphate-sulfite reductase of enterobacteria. 3. The *Escherichia coli* hemoflavoprotein: catalytic parameters and the sequence of electron flow. J Biol Chem 249:1572-86.

16. Clarke TA, Mills PC, Poock SR, Butt JN, Cheesman MR, Cole JA, Hinton JCD, Hemmings AM, Kemp G, Söderberg CAG, Spiro S, Van Wonderen J, Richardson DJ. 2008. *Escherichia coli* Cytochrome c Nitrite Reductase NrfA. Methods Enzymol 437:63-77.
17. Jackson RH, Cole JA, Cornish-Bowden A. 1982. The steady state kinetics of the NADH-dependent nitrite reductase from *Escherichia coli* K12. The reduction of single-electron acceptors. Biochemical Journal 203:505-510.
18. Krueger RJ, Siegel LM. 1982. Spinach siroheme enzymes: isolation and characterization of ferredoxin-sulfite reductase and comparison of properties with ferredoxin-nitrite reductase. Biochemistry 21:2892-2904.
19. Pereira IC, Abreu IA, Xavier AV, LeGall J, Teixeira M. 1996. Nitrite Reductase from *Desulfovibrio desulfuricans* (ATCC 27774) — A Heterooligomer Heme Protein with Sulfite Reductase Activity. Biochemical and Biophysical Research Communications 224:611-618.
20. Mohn M, Thaqi B, Fischer-Schrader K. 2019. Isoform-Specific NO Synthesis by *Arabidopsis thaliana* Nitrate Reductase. Plants 8:67.
21. Guerrero-Cruz S, Stultiens K, van Kessel M, Versantvoort W, Jetten MSM, Op den Camp HJM, Kartal B. 2019. Key Physiology of a Nitrite-Dependent Methane-Oxidizing Enrichment Culture. Appl Environ Microbiol 85.
22. Martínez-Espinosa RM, Marhuenda-Egea FC, Bonete MJ. 2001. Purification and characterisation of a possible assimilatory nitrite reductase from the halophile archaeon *Haloferax mediterranei*. FEMS Microbiology Letters 196:113-118.
23. Kobayashi S, Hira D, Yoshida K, Toyofuku M, Shida Y, Ogasawara W, Yamaguchi T, Araki N, Oshiki M. 2018. Nitric Oxide Production from Nitrite Reduction and Hydroxylamine Oxidation by Copper-containing Dissimilatory Nitrite Reductase (NirK) from the Aerobic Ammonia-oxidizing Archaeon, *Nitrososphaera viennensis*. Microbes and Environments 33:428-434.
